# Altered hepatic lipid droplet morphology and lipid metabolism in fasted Plin2-null mice

**DOI:** 10.1016/j.jlr.2023.100461

**Published:** 2023-10-14

**Authors:** Atanaska I. Doncheva, Yuchuan Li, Prabhat Khanal, Marit Hjorth, Svein O. Kolset, Frode A. Norheim, Alan R. Kimmel, Knut Tomas Dalen

**Affiliations:** 1 Department of Nutrition, Institute of Basic Medical Sciences, Faculty of Medicine, University of Oslo, Oslo, Norway; 2 Department of Hepato-Pancreato-Biliary Surgery, Institute of Clinical Medicine, University of Oslo, Oslo, Norway; 3 Faculty of Biosciences and Aquaculture, Nord University, Steinkjer, Norway; 4 Laboratory of Cellular and Developmental Biology, National Institute of Diabetes and Digestive and Kidney Diseases, The National Institutes of Health, Bethesda, MD, USA; 5 The Norwegian Transgenic Center, Institute of Basic Medical Sciences, University of Oslo, Oslo, Norway

**Keywords:** Plin2, liver, triglyceride, cholesteryl ester, lipid droplets, fasting

## Abstract

Perilipin 2 (Plin2) binds to the surface of hepatic lipid droplets (LDs) with expression levels that correlate with triacylglyceride (TAG) content. We investigated if Plin2 is important for hepatic LD storage in fasted or high-fat diet-induced obese *Plin2*^+/+^ and *Plin2*^−/−^ mice. *Plin2*^−/−^ mice had comparable body weights, metabolic phenotype, glucose tolerance, and circulating TAG and total cholesterol levels compared with *Plin2*^+/+^ mice, regardless of the dietary regime. Both fasted and high-fat fed *Plin2*^−/−^ mice stored reduced levels of hepatic TAG compared with *Plin2*^+/+^ mice. Fasted *Plin2*^−/−^ mice stored fewer but larger hepatic LDs compared with *Plin2*^+/+^ mice. Detailed hepatic lipid analysis showed substantial reductions in accumulated TAG species in fasted *Plin2*^−/−^ mice compared with *Plin2*^+/+^ mice, whereas cholesteryl esters and phosphatidylcholines were increased. RNA-Seq revealed minor differences in hepatic gene expression between fed *Plin2*^+/+^ and *Plin2*^−/−^ mice, in contrast to marked differences in gene expression between fasted *Plin2*^+/+^ and *Plin2*^−/−^ mice. Our findings demonstrate that Plin2 is required to regulate hepatic LD size and storage of neutral lipid species in the fasted state, while its role in obesity-induced steatosis is less clear.

Lipids constitute a diverse group of organic molecules that are important for cellular membrane synthesis, cell signaling, organelle interactions, energy metabolism, and energy storage (1, 2). Because of their hydrophobic properties, most lipids are poorly soluble in the cytosol and are either incorporated into cellular membranes or stored in lipid droplet (LD) organelles. In mammalian cells, the central LD core mainly consists of neutral lipids such as triacylglycerols (TAGs) and/or cholesteryl esters (CEs), although certain specialized cell types may store other esterified lipids, such as retinyl esters (1). The LD surface is surrounded by a monolayer of phospholipids and a cell-specific population of LD-associated proteins, such as the perilipins. Perilipins exhibit very high sequence similarity and have evolutionary and functional relationships that can be traced for more than 500 million years (2, 3). The perilipin family comprises five genes (*Plin1-5*) in mammals, and their alternative splicing can generate at least 10 different protein isoforms in mice (4). A standardized Perilipin/Plin nomenclature has been introduced (5) for these tissue-specific proteins that interact with lipases and the lipophagy machinery at the LD surface (6).

One of the first mammalian proteins detected at the surface of cytosolic LDs is perilipin 1 (Plin1) (7). Expression of Plin1 is restricted to adipocytes and steroid cells (7). Perilipin 2 (Plin2), initially named adipose differentiation-related protein/ADRP (8), adipophilin (9), and ADFP (10), is the second family member confirmed to bind to LDs (11). Plin2 is unstable and rapidly ubiquitinylated and degraded by the proteasome when not bound to LDs (12, 13). Plin1 seems able to displace Plin2 from the LD surface. Consequently, Plin2 proteins are not detected in adipocytes expressing Plin1 despite the high presence of *Plin2* mRNA (11), perhaps except in lipolytically stimulated adipocytes where Plin2 proteins may be stabilized upon phosphorylation of Plin1 (14). Plin2 is ubiquitously expressed (11, 15), but its expression can be highly elevated when cells are exposed to FAs (16, 17). Plin3 is ubiquitously expressed (15) and the protein is stable in the cytosol independent of intracellular LD levels (18). Plin4 contains a divergent extended central region compared with other Plins and is the least studied family member (19). Plin3 and Plin4 may coat LDs during LD growth when the amounts of other Plin proteins are insufficient to coat expanding phospholipid surfaces (18, 20). Plin5 is primarily expressed in oxidative cells, for example, heart and muscle (19, 21), and is involved in the shuttling of FAs from LDs to mitochondria to maintain mitochondrial integrity (22, 23).

Neutral lipids stored in LDs can be mobilized via lipolytic degradation by cytosolic neutral lipases, alternatively by lipophagy in a process where LDs are engulfed by phagophores and degraded in lysosomes (24). Plin proteins are involved in both processes, but their role in lipolysis is better characterized. In the basal state, nonphosphorylated Plin1 protects TAG stores from lipolytic degradation in white adipose tissue (25), whereas Plin5 has a similar role in tissues with high oxidative capacity (e.g., heart and brown adipose tissue) (26, 27). Upon lipolytic stimulation, Plin1 is phosphorylated (2, 7, 28) and enhances lipolysis by releasing abhydrolase domain containing 5 (ABHD5), enabling this lipase coactivator to bind and activate adipose triglyceride lipase (ATGL) at the LD surface. Phosphorylation of Plin5 will also stimulate lipolysis (29), but since Plin5 binds to both ATGL (29) and Abhd5 (30), Plin5 is likely to regulate ATGL activity differently than Plin1. The first and rate-limiting step of TAG lipolysis in adipocytes is activated by ATGL hydrolyzing TAG into diacylglycerol (DAG) and FFA. Subsequently, DAG is cleaved by hormone-sensitive lipase (HSL), followed by cleavage of monoacylglycerol by monoacylglycerol lipase into FFAs and glycerol (31). The importance of Plin proteins and the lipolytic pathway are less defined in the liver. The liver expresses predominantly Plin2 and Plin3, with lower expression of several key lipolytic factors, such as Plin4, Plin5, and HSL (6, 32). Removal of Plin2 depletes TAG stores in the liver (10), but it remains to be determined if Plin2 and Plin3 bind and immobilize ATGL at the LD surface similar to Plin1 and Plin5.

Accumulation of LDs in the liver and other metabolically active tissues is strongly correlated to obesity-mediated metabolic disorders and insulin resistance (33). It is not fully clarified whether liver steatosis protects against or promotes the development of insulin resistance or if LDs accumulate as a consequence of insulin resistance. Hepatic expression of Plin2 is enhanced in obese mice (34, 35) and humans with fatty liver disease (35). In line with these observations, hepatic overexpression of Plin2 in mice increases lipid accumulation (36, 37), whereas global (10, 38, 39) or liver-specific removal of Plin2 (38, 40) reduces hepatic lipid storage. Removal of Plin2 may also protect against the development of hepatic insulin resistance (38, 39, 41). In contrast, muscular overexpression of Plin2 seems to enhance insulin sensitivity despite increased lipid accumulation (42), implying that the consequences of LD deposition can vary between tissues.

Although obesity and insulin resistance promote TAG accumulation and hepatic steatosis, prolonged energy deprivation has a stronger impact on hepatic TAG levels. Fasting triggers a massive release of FAs from adipose tissue into the circulation, promoting FA uptake into multiple nonadipocyte organs, including the liver. In response to overnight fasting, hepatic expression of *Plin2* and *Plin5* mRNAs and proteins increase substantially (17, 43). While Plin5 may help to limit hepatic inflammation during fasting (43), the role of hepatic Plin2 during fasting is unclear. In this study, we examined the role of Plin2 in the coating of hepatic LDs by analyzing livers of fasted and diet-induced obese *Plin2*^+/+^ and *Plin2*^−/−^ mice.

## Materials and Methods

### Ethics and animal housing conditions

All experimental uses of animals were approved and registered by the Norwegian Animal Research Authority (Mattilsynet, approval FOTS IDs: #10901, #10902, and #24912) and conformed to the ARRIVE (Animal Research: Reporting of In Vivo Experiments) guidelines and ethical guidelines in Directive 2010/63/EU of the European Parliament on the protection of animals used for scientific purposes. All mice were housed with a stable light/dark cycle (07 AM–07 PM), with 55 ± 5% relative humidity at 22 ± 2°C in a specific pathogen-free animal unit. Mice had free access to water and rodent chow or custom-made diets. The presence of pathogens was monitored quarterly in accordance with FELASA (Federation of European Laboratory Animal Science Associations) recommendations without detection of any screened pathogens.

### Mice diet interventions

Generation of *Plin2*^−/−^ mice with disruption of Plin2 exons 4–6 has been described previously (44). All *Plin2*^−/−^ mice were back-crossed into C57BL/6N for >10 generations prior to experiments. *Plin2*^+/+^ and *Plin2*^−/−^ mice were euthanized by cervical dislocation, followed by a rapid collection of tissues, which were snap-frozen in liquid nitrogen. In total, 116 mice were used.

#### Fasting intervention

Female *Plin2*^+/+^ and *Plin2*^−/−^ mice (15 weeks of age) from the same breeding colony were housed with free access to standard chow (58 E% carbohydrate, 18 E% fat, 24 E% protein; catalog no.: 2018SX, Envigo, IN) or fasted for 24 h. Chow-fed mice remained in their original cages, whereas mice under fasting were transferred in pairs to clean cages equipped with a water bottle and a plastic shelter but no bedding material or eatable items. In total, 38 mice were included in the study (20 *Plin2*^+/+^ and 18 *Plin2*^−/−^ mice). None of the included animals were excluded from the analyses.

#### High-fat diet intervention

Female *Plin2*^+/+^ and *Plin2*^−/−^ animals were reproduced and housed with free access to rodent chow (62 E% carbohydrate, 11 E% fat, 27 E% protein; catalog no.: RM3A/SDS RM3, Scanbur, Denmark). Mice were given either a low-fat diet (LFD; 3.85 kcal/g, 70 E% from carbohydrates, 10 E% from fat, and 20 E% from protein, catalog no.: D12450J, Research Diets, New Brunswick, NJ) or a high-fat diet (HFD; 5.24 kcal/g, 20 E% from carbohydrates, 60 E% from fat, and 20 E% from protein, catalog no.: D12492J, Research Diets) from 8 weeks of age until 18 or 28 weeks of age (10 or 20 weeks of diet intervention). The two diets differed only in calorie density and their content of carbohydrates and saturated fat. *Plin2*^+/+^ and *Plin2*^−/−^ animals were randomly allocated in groups of 4 ± 1 mice per cage (both genotypes represented in each cage) to minimize microbiota variations. Body weights were measured weekly during the midportion of the light cycle. In total, 78 mice were included in the study (41 *Plin2*^+/+^ and 37 *Plin2*^−/−^ mice). None of the included animals were excluded from the analyses.

### Mice phenotyping procedures

#### Body composition

Body composition was determined at 18 weeks of age (10 weeks of diet) and 28 weeks of age (20 weeks of diet) with a Minispec LF90II magnetic resonance imaging machine (Bruker, Billerica, MA).

#### Oral glucose tolerance test

Oral glucose tolerance test was performed at 17 weeks of age (9 weeks of diet) and 27 weeks of age (19 weeks of diet). Briefly, after a fasting period of 5 h (9–14 AM), glucose (d-(+)-glucose, catalog no.: G7021; Sigma, Darmstadt, Germany) was administered via oral gavage dosed at 1.5 g/kg lean mass. Blood glucose was measured with a glucose meter (FreeStyle Precision Neo, Abbot, The Netherlands) in whole blood collected from the tip of the tail at 0, 15, 30, 60, 90, and 120 min time points. Insulin levels during the glucose tolerance test were determined in serum collected at 0, 15, and 30 min time points using an Ultra Sensitive Mouse Insulin ELISA kit (catalog no.: 90080, Crystal Chem, Elk Grove Village, IL).

#### Indirect calorimetry

A week before euthanasia, mice were placed in an indirect calorimetry system consisting of 20 individual cages (PhenoMaster, TSE Systems, Germany). Indirect calorimetric measurements were performed as described previously (45). Gas flow was 0.42 l/min, with gas exchange measured for 10 s per cage in 20 min intervals. Physical activity was recorded as movements in the XY plane. Food intake was calculated for individual mice by measuring diet before and after indirect calorimetry measurements.

### Isolation of total RNA and RT-qPCR

Total RNA was isolated with NucleoSpin RNA kit (Macherey-Nagel, GmbH & Co KG, Düren, Germany), with a modified protocol described previously (44). RNA was eluted in 40 μl RNase-free water and stored at −80°C. Concentrations and purity of RNA samples were determined with NanoDrop ND-1000 Spectrophotometer (Thermo Fisher Scientific, Wilmington).

Total RNA (12.5 ng/μl) was reversely transcribed with random hexamers and the High Capacity cDNA Reverse Transcription Kit (catalog no.: 4368814, Thermo Fisher Scientific) on an Eppendorf Mastercycler EP Gradient S (Eppendorf AG, Hamburg, Germany) as described previously (44). Gene-specific regions were amplified from 10 ng/μl complementary DNA with gene-specific assay primers (200 nmol/l each, listed in supplemental Table S1) and Bio-Rad SsoAdvanced™ Universal SYBR® Green Supermix using the Bio-Rad CFX96 system (Bio-Rad, Hercules, CA). Cycling conditions and primer design have been described previously (44). Data are presented as gene expression levels relative to the expression of *Tbp* (2−^ΔCq^).

### RNA-Seq and read counting

The total RNA quantity and integrity were measured with a Bioanalyzer RNA 6000 Nano Kit (Agilent Technologies, Santa Clara, CA). The RNA samples had RIN values between 7.6 and 8.9. Total RNA samples were subjected to Strand-Specific TruSeq^TM^ mRNA-Seq library preparation, and 50 bp paired-end reads were sequenced at the Norwegian Sequencing Centre (Oslo University Hospital, Ullevål, Oslo) as described before (45) on an Illumina Novaseq 600 instrument. BBDuk (BBMap v34.56 was used to remove/trim low-quality reads and adapter sequences sourceforge.net/projects/bbmap/). Reads were aligned against the *Mus musculus* reference genome (ENSEMBL release 101, GRCh38.101) using HiSat2, version 1.2.1 (46). FeatureCounts v1.4.6-p1 (http://subread.sourceforge.net) was used for read counting (47), with an average read of 43 millions/sample. Raw sequencing data and normalized counts are available in the National Center for Biotechnology Information Gene Expression Omnibus repository (GSE214064).

Differential RNA expression analyses were performed in R, version 4.0.3 software with the EdgeR package (48). Graphs were generated using the EnhancedVolcano package (https://github.com/kevinblighe/EnhancedVolcano). A false discovery rate (FDR) of 5% was used as the threshold for statistical significance. Gene Ontology analyses were performed with the clusterProfiler package (49) with Kyoto Encyclopedia of Genes and Genomes (KEGG) pathway annotations using the default parameters: organism(mmu), keyType(keg), pvalueCutoff(0.05), pAdjustMethod(BH), minGSSize(20), maxGSSize(500), and qvalueCutoff(0.05).

### Immunoblotting

Liver tissue was lysed in radioimmunoprecipitation assay buffer containing cOmplete Proteinase Inhibitor Cocktail (catalog no.: 11836170001; Roche, Basel, Switzerland) and phosphatase inhibitors (catalog no.: P0044; Sigma-Aldrich, St Louis, MO). Samples were homogenized with glass beads in a Precellys 24 tissue homogenizer (Bertin Instruments, Montigny-le-Bretonneux, France) and sonicated on a Bioruptor® Plus device (Diagenode, Liege, Belgium). Lysates were mixed with Laemmli buffer, separated on Criterion™ TGX™ 4–20% gels (Bio-Rad, Hercules, CA), and transferred to nitrocellulose membranes using the RTA transfer kit and Trans-Blot Turbo transfer system (Bio-Rad, Hercules, CA). Membranes were stained with Ponceau S to determine the total protein loaded in each lane. Primary antibodies used were guinea pig anti-Plin2 (catalog no.: GP-40; Progen, Heidelberg, Germany), rabbit anti-Lamp1 (catalog no.: 3243; Cell Signaling, Danvers, MA), rabbit anti-light chain 3 B (LC3B) (catalog no.: 2775; Cell Signaling), rabbit anti-SQSTM1/p62 (catalog no.: 5114; Cell Signaling), rabbit anti-PEX14 (catalog no.: ABC142; MilliporeSigma, MA), rat anti-Lamp2 (catalog no.: ab13524; Abcam, Cambridge, UK), guinea anti-Plin3 (catalog no.: GP-40; Progen), guinea anti-Plin5 (catalog no.: GP-44; Progen), anti-rabbit HSL (catalog no.: 4107; Cell Signaling), anti-rabbit ATGL (catalog no.: 2439S; Cell Signaling), and anti-rabbit phosphorylated HSL (p-HSL) Ser565 (catalog no.: 4137S; Cell Signaling). Secondary antibodies used were goat anti-rabbit IgG (catalog no.: 111-035-144), donkey anti-guinea pig IgG (catalog no.: 706-035-148; Jackson ImmunoResearch, West Grove, PA), and goat anti-rat IgG (catalog no.: AP136P; Sigma-Aldrich, Steinheim, Germany). Chemiluminescence detection was done on a ChemiDoc™ Touch Imaging System (Bio-Rad, Hercules, CA). Band intensities were quantified using ImageJ, version 1.52 software (National Institutes of Health, Bethesda, MD), with protein abundances given relative to total protein levels (Ponceau signal) in the corresponding lane.

### Liver histology

Liver tissues were fixed for ∼24 h at 4°C in 4% paraformaldehyde in 0.1 mol/l phosphate buffer (PB; pH 7.4) (4% paraformaldehyde-PB) and subsequently stored at 4°C in 0.4% paraformaldehyde-PB until processing. Liver tissues were cryoprotected with a gradient of sucrose solved in 0.1 mol/l PB and then frozen in optimal cutting temperature embedding matrix (KMA-0100-00A; CellPath, Newtown, UK) over nitrogen vapor in embedding molds (E4140-1EA; Sigma-Aldrich, Steinheim, Germany) as described previously (44). Sections (20 μm) were prepared from optimal cutting temperature blocks with a Leica CM3050S cryostat (Leica Biosystems, Deer Park, IL) at −20°C.

Sections were washed in PB and stained for 25 min while floating in PB containing 1 μmol/l Bodipy 493/503 (Thermo Fisher Scientific, Waltham, MA), 5 μmol/l Hoechst-33342 (Sigma-Aldrich, St Louis, MO), and 1 U/ml CF568 conjugated phalloidin (Biotium, Fremont, CA). After staining, sections were washed two times in PB and then mounted on SuperFrost Plus slides (VWR, Radnor, PA) with ProLong mounting media (Thermo Fisher Scientific, Waltham, MA) and coverslips. The slides were allowed to harden overnight at room temperature and stored at 4°C in the dark. All steps from sectioning until confocal imaging were completed within 2 weeks.

#### Confocal microscopy

High-resolution confocal images were taken under a 40× oil immersion objective mounted on an LSM 710 confocal microscope (Zeiss) as described previously (44). Laser and detection settings: nucleus staining (excited with a 405 nm laser/fluorescence detected at 414–465 nm, LD staining [488 nm/497–545 nm], and cell skeleton staining [561 nm/563–632 nm]). Signal intensities were optimized for each channel with a representative section and reused for the remaining sections.

#### Quantification of LDs

Confocal images were used for LD quantification using ImageJ, version 1.54 software. The diameters of individual LDs were manually measured for one representative image per mouse (*n* = 5 mice in each group). The number of LDs measured on each image ranged from 121 to 336 LDs (average number = ∼200 LDs per image). The corresponding numbers of nuclei were counted on the same images and used for normalization.

### Hepatic and plasma TAG, total cholesterol, and ketone bodies content

Lipids were measured in plasma and homogenates of liver tissue using colorimetric enzymatic detection kits for detection of total TAG (catalog no.: T75321L; Pointe Scientific, Canton, MI), total cholesterol (Chol; catalog no.: C75101L; Pointe Scientific, Canton, MI), and ketone bodies (catalog no.: MAK134; Sigma-Aldrich, St Louis, MO) according to the manufacturer's instructions.

### Detailed lipid analysis

Around 100 mg of liver tissue was used for lipid analysis using HPLC coupled with time-of-flight mass spectrometry as previously described (44). The following lipid species were measured: cardiolipins (CLs), ceramides (Cers), Chol, CEs, DAG, ether-linked phosphatidylcholine (PCO), FFAs, hexosylceramide (HexCer), lysophosphatidylcholine, lysophosphatidylethanolamine (LPE), bis(monoacylglycerol)phosphate, phosphatidylcholine (PC), phosphatidylcholine plasmalogen, phosphatidylethanolamine (PE), phosphatidylethanolamine plasmalogen, phosphatidylglycerol (PG), phosphatidylinositol (PI), phosphatidylserine (PS), sphingomyelin (SM), and TAG. Detected lipid signals were normalized against liver weights and internal standards. Internal standards used were CL (C56:0), Cer (C35:1), Chold47, CE (C19:0), DAG (C30:0), FFA (C17:1), HexCer (C35:1), lysophosphatidylcholine (C17:1), LPE (C17:1), PC (C28:0), PE (C28:0), PG (C30:0), PI (C31:1), PS (C28:0), SM (C35:1), and TAG (C39:0), all from Sigma-Aldrich (St Louis, MO).

### Statistics

Data were analyzed with Prism 5 (GraphPad Software, CA). Two-way ANOVA with Tukey post hoc tests and multiple *t*-tests were used where *P* < 0.05 were considered significant. Data in graphs are shown as means ± 95% confidence interval.

## Results

### *Plin2*^−/−^ mice have normal whole body and organ weights when fasted

Parallel studies in alternatively generated *Plin2*-null models have established that loss of Plin2 results in reduced hepatic lipid levels in fed mice (10, 50). We showed previously that fasting in mice initiates massive accumulation of hepatic LDs, accompanied by upregulation of *Plin2* mRNA and protein levels (17). To investigate the importance of Plin2 for hepatic LD formation and lipid metabolism during fasting, 15-week-old female and male *Plin2*^+/+^ and *Plin2*^−/−^ mice were given free access to a chow diet (fed) or were fasted for 24 h (fasted). As expected, body weights, and liver, gonadal, and subcutaneous white adipose tissue weights were reduced in both *Plin2*^+/+^ and *Plin2*^−/−^ mice after 24 h of fasting (Table 1). Heart and kidney weights were unaffected by fasting. Except for slightly higher liver weights in chow-fed female *Plin2*^−/−^ mice and lower heart weight in fasted male *Plin2*^+/+^ mice, body weights and organ weights were essentially similar in *Plin2*^+/+^ and *Plin2*^−/−^ mice.

**Table 1:** Body weights and organ weights of 15-week-old *Plin2*^+/+^ and *Plin2*^−/−^ mice with ad libitum access to chow diet or after 24 h of fasting

Organs	Fed		Fasted (24 h)	
	*Plin2*^+/+^	*Plin2*^−/−^	*Plin2*^+/+^	*Plin2*^−/−^
15 weeks—males				
Body weight	28.1 ± 1.4	28.1 ± 2.4	22.7 ± 2.1^d^	24.5 ± 2.5^d^
Liver (g)	1.45 ± 0.09	1.37 ± 0.16	0.97 ± 0.09^d^	0.97 ± 0.12^d^
Heart (g)	0.128 ± 0.011	0.129 ± 0.008	0.117 ± 0.009	0.132 ± 0.015^a^
Kidney (g)	0.34 ± 0.05	0.34 ± 0.04	0.31 ± 0.04	0.33 ± 0.06
White adipose tissue epididymal (g)	0.25 ± 0.05	0.21 ± 0.05	0.14 ± 0.11^c^	0.16 ± 0.07
White adipose tissue subcutaneous (g)	0.16 ± 0.03	0.16 ± 0.03	0.09 ± 0.06^c^	0.08 ± 0.04^c^
15 weeks—females				
Body weight (g)	21.5 ± 0.9	22.4 ± 1.2	18.0 ± 1.1^d^	18.7 ± 1.4^d^
Liver (g)	0.92 ± 0.08	1.03 ± 0.10^a^	0.82 ± 0.08^b^	0.76 ± 0.1^d^
Heart (g)	0.094 ± 0.009	0.093 ± 0.007	0.096 ± 0.006	0.092 ± 0.009
Kidney (g)	0.23 ± 0.02	0.23 ± 0.02	0.24 ± 0.02	0.23 ± 0.03
White adipose tissue gonadal (g)	0.21 ± 0.10	0.19 ± 0.09	0.09 ± 0.05^b^	0.11 ± 0.06
White adipose tissue subcutaneous (g)	0.17 ± 0.04	0.17 ± 0.05	0.08 ± 0.02^d^	0.09 ± 0.03^d^

Data are presented as means ± SD, *n* = 8–10.

a *P* < 0.05 indicates difference between *Plin2*^+/+^ and *Plin2*^−/−^ mice with the same feeding condition.

b *P* < 0.05,

c *P* < 0.01, and

d *P* < 0.001 indicates difference between fed and fasted mice of the same genotype.

### Plin3 and Plin5 proteins increase in the liver of fasted *Plin2*^−/−^ mice

**Fig. 1:**
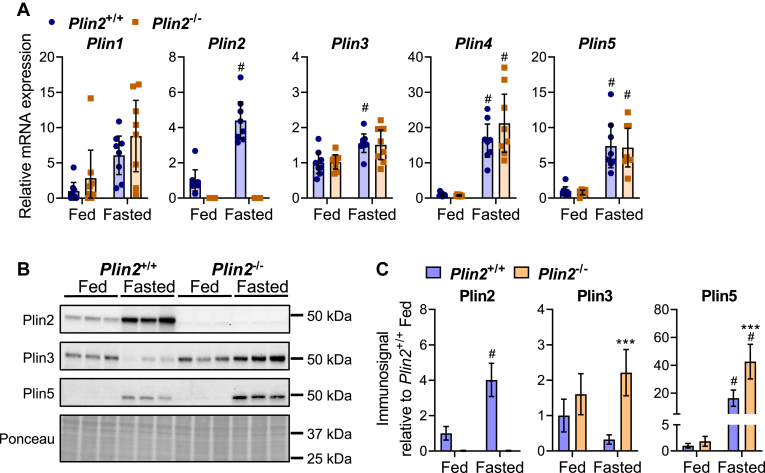
Hepatic expression of Plins in fed and fasted *Plin2*^+/+^ and *Plin2*^−/−^ mice. Female *Plin2*^+/+^ and *Plin2*^−/−^ mice (15 weeks) were housed with ad libitum access to a chow diet (fed) or were fasted for 24 h (fasted) prior to euthanasia at 8–10 AM (*n* = 8 per group). A: Relative expression of *Plin1*, *Plin2*, *Plin3*, *Plin4*, and *Plin5* mRNAs in liver. B: Representative immunoblots showing expression of Plin2, Plin3, and Plin5 proteins in three individual animals. C: Relative quantification of hepatic Plin protein expression levels (*n* = 6). Statistical testing was performed with two-way ANOVA and Tukey test for multiple comparisons. #*P* < 0.05 indicates the statistical difference between fed and fasted conditions for the same genotype; ∗∗∗*P* < 0.001 indicates the difference between *Plin2*^+/+^ and *Plin2*^−/−^ mice with the same feeding condition. Data in graphs are shown as means ± 95% confidence interval.

Upon loss of a Plin, other Plin proteins are known to compensate and bind to the LD surface (2). To determine if deletion of *Plin2* altered the expression of other Plins, we measured *Plin1-5* mRNAs in the liver. Fasting increased hepatic expression of *Plin2*, *Plin4*, and *Plin5* mRNAs substantially (>5-fold), whereas expression of *Plin3* mRNA was modestly increased (Fig. 1A). Expression of Plin1 was low and exhibited higher variation between individuals compared with *Plin2-Plin5* mRNAs. Except for the anticipated absence of *Plin2* mRNA in *Plin2*^*−/−*^ mice, the remaining *Plin* mRNAs were expressed at similar levels in *Plin2*^+/+^ and *Plin2*^−/−^ mice. Several Plins are subjected to post-translational regulation (2), and their mRNA levels do not reliably predict their protein expression levels. We therefore measured protein levels of the abundantly expressed perilipin proteins; Plin2, Plin3, and Plin5 with immunoblotting. Consistent with previous findings, Plin2 protein levels increased in the livers of *Plin2*^+/+^ mice upon fasting (17), whereas Plin2 protein was not detected in fed or fasted *Plin2*^−/−^ mice (Fig. 1B). Whole-cell Plin3 protein levels were similar in fed *Plin2*^+/+^ and *Plin2*^−/−^ mice but increased in fasted *Plin2*^−/−^ mice and tended to decrease in fasted *Plin2*^+/+^ mice, resulting in higher Plin3 protein levels in fasted *Plin2*^−/−^ mice compared with *Plin2*^+/+^ mice (Fig. 1C). Plin5 protein levels were similar in fed *Plin2*^+/+^ and *Plin2*^−/−^ mice but substantially increased with fasting, reaching significantly higher levels in fasted *Plin2*^−/−^ mice compared with *Plin2*^+/+^ mice. Hence, both Plin3 and Plin5 proteins were increased in fasted *Plin2*^−/−^ mice, suggesting they compensate for the hepatic loss of Plin2.

### Fasted *Plin2*^−/−^ mice have decreased hepatic TAG content but store LDs with increased size

**Fig. 2:**
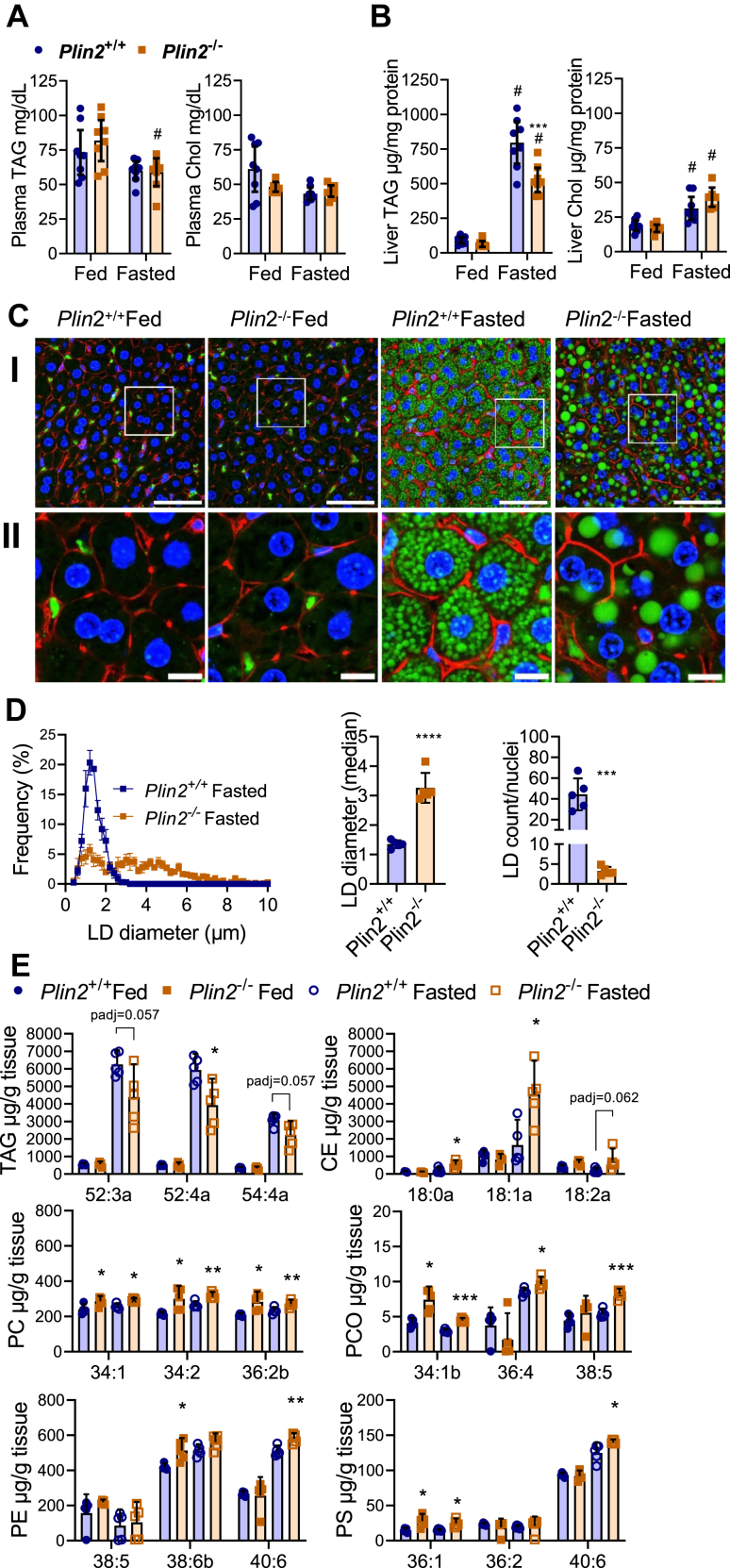
Lipids abundance in fed and fasted *Plin2*^+/+^ and *Plin2*^−/−^ mice. Female *Plin2*^+/+^ and *Plin2*^−/−^ mice (15 weeks) were housed with ad libitum access to a chow diet (fed) or were fasted for 24 h (fasted) prior to euthanasia at 8–10 AM (*n* = 8 per group). A: Plasma TAGs and total Chol levels. B: Liver TAG and Chol levels. Statistical testing was performed with two-way ANOVA and Tukey test for multiple comparisons. #*P* < 0.05 indicates statistical difference between fed and fasted condition; ∗∗∗*P* < 0.001 difference between *Plin2*^+/+^ and *Plin2*^−/−^ mice with the same feeding condition. C: Representative images of liver cryosections. Cryosections (20 μm thick) were stained to visualize LDs (Bodipy 493/503, green), nuclei (Hoechst 33342, blue), and plasma membrane-located F-actin (Phalloidin-CF568, red). I: Confocal images of liver sections taken with a 40× objective. II: Pictures zoomed in from I to visualize individual cells (inserted boxes indicate enlarged areas). Scale bars represent 50 μm (I) and 10 μm (II). D: Size distribution of hepatic LDs, LD diameter, and LD numbers in fasted *Plin2*^+/+^ and *Plin2*^−/−^ mice (*n* = 5 in each group). LD numbers were normalized against nuclei. E: Analysis of selected hepatic lipid species with HPLC-qTOF/MS: TAG, CE, PC, PCO, PE, and PS species (*n* = 5 per group). Statistical testing was performed with multiple *t*-tests between *Plin2*^+/+^ and *Plin2*^−/−^ mice in fed or fasted states. ∗*P* < 0.05, ∗∗*P* < 0.01, and ∗∗∗*P* < 0.001. Data in graphs are shown as means ± 95% confidence interval.

Several studies have reported a reduction in hepatic lipids upon loss of Plin2 in fed conditions (10, 38, 39). To compare lipid levels in fed and fasted conditions, we examined plasma and hepatic TAG and total Chol content in *Plin2*^+/+^ and *Plin2*^−/−^ mice. Plasma TAG and total Chol levels were similar in *Plin2*^+/+^ and *Plin2*^−/−^ mice and remained essentially unaltered by fasting (Fig. 2A). Hepatic TAG levels were similar in fed *Plin2*^+/+^ and *Plin2*^−/−^ mice, whereas levels were massively increased by fasting but less in fasted *Plin2*^−/−^ mice compared with fasted *Plin2*^+/+^ mice (Fig. 2B). Hepatic total Chol increased with fasting with a trend for higher levels in fasted *Plin2*^−/−^ mice compared with *Plin2*^+/+^ mice (*P* = 0.056).

To follow up on differences in hepatic TAG content in fasted mice, we examined hepatic LD content and histological structures in fed and fasted *Plin2*^+/+^ and *Plin2*^−/−^ mice. Sections were triple stained to visualize LDs (Bodipy 493/503, green), nuclei (Hoechst 33342, blue), and plasma membrane-located F-actin (Phalloidin-CF568, red). In consistency with measured TAG levels, LD levels (green staining) were comparable at low levels in fed *Plin2*^+/+^ and *Plin2*^−/−^ mice (Fig. 2C). In contrast, a massive increase in LD levels was observed in fasted mice (Fig. 2C) but with LDs that were distinctly different between *Plin2*^+/+^ and *Plin2*^−/−^ mice. Hepatic LDs in fasted *Plin2*^+/+^ mice were small, numerous, and dispersed in the cytoplasm of the lipid-containing hepatocytes, whereas a large majority of LDs in fasted *Plin2*^−/−^ mice were typically much larger in size compared with LDs observed in fasted *Plin2*^+/+^ mice (Fig. 2D). Quantification of LDs in the fasted state demonstrated a clear reduction in the LD number and an increase in the LD size in fasted *Plin2*^−/−^ mice compared with fasted *Plin2*^+/+^ mice (Fig. 2D). Because of the considerably smaller size of LDs in the fed state, we were not able to accurately quantify the size and number of those LDs.

To gain insights into differences in hepatic lipid species between *Plin2*^+/+^ and *Plin2*^−/−^ mice, we performed detailed lipid analysis with HPLC-qTOF/MS. Various species of TAG (number of carbons:double bounds ranging from TAG48:1a to TAG58:10a), DAG species (DAG32:0 to DAG40:6), and FFAs (16:0 to 22:6) tended consistently to be less abundant in livers of fed *Plin2*^−/−^ mice compared with fed *Plin2*^+/+^ mice (supplemental Fig. S1). Upon fasting, various species of TAG, DAG, and PE, as well as most FFAs, were increased, whereof TAG and DAG seem to be robustly increased and represent the major hepatic lipids accumulating in the liver upon fasting (supplemental Fig. S1). The three most abundant TAG species (Fig. 2E) and the majority of other TAG species were less abundant in the livers of fasted *Plin2*^−/−^ mice compared with fasted *Plin2*^+/+^ mice (supplemental Fig. S1). All measured CE species were elevated in the livers of fasted *Plin2*^−/−^ mice compared with fasted *Plin2*^+/+^ mice (Fig. 2C and supplemental Fig. S1), and CE species were differently affected by fasting based on their FA chain length. CE species consisting of saturated/monounsaturated FAs (CE16:0 to CE18:1) increased by fasting, whereas CE species consisting of longer polyunsaturated FAs (CE18:2 to CE22:6) decreased by fasting (supplemental Fig. S1). For the other measured lipids, the abundance of PC, PCO, and PS species tended to be higher in fed or fasted *Plin2*^−/−^ compared with *Plin2*^+/+^ mice (Fig. 2D, and supplemental Fig. S1). Levels of some PE species were also elevated in *Plin2*^−/−^ mice (Fig. 2E and supplemental Fig. S2). Bis(monoacylglycerol)phosphate and HexCer seemed to be higher in fed *Plin2*^−/−^ mice compared with *Plin2*^+/+^ mice, though these lipid species were found at low levels affecting the accuracy of their quantification. The abundance of other lipids like Cer, LCP, LPE, PG, PI, SM, and CL in the liver was essentially similar between *Plin2*^+/+^ and *Plin2*^−/−^ mice (supplemental Fig. S1).

### Hepatic gene expression differs between fasted *Plin2*^+/+^ and *Plin2*^*−*/−^ mice

**Fig. 3:**
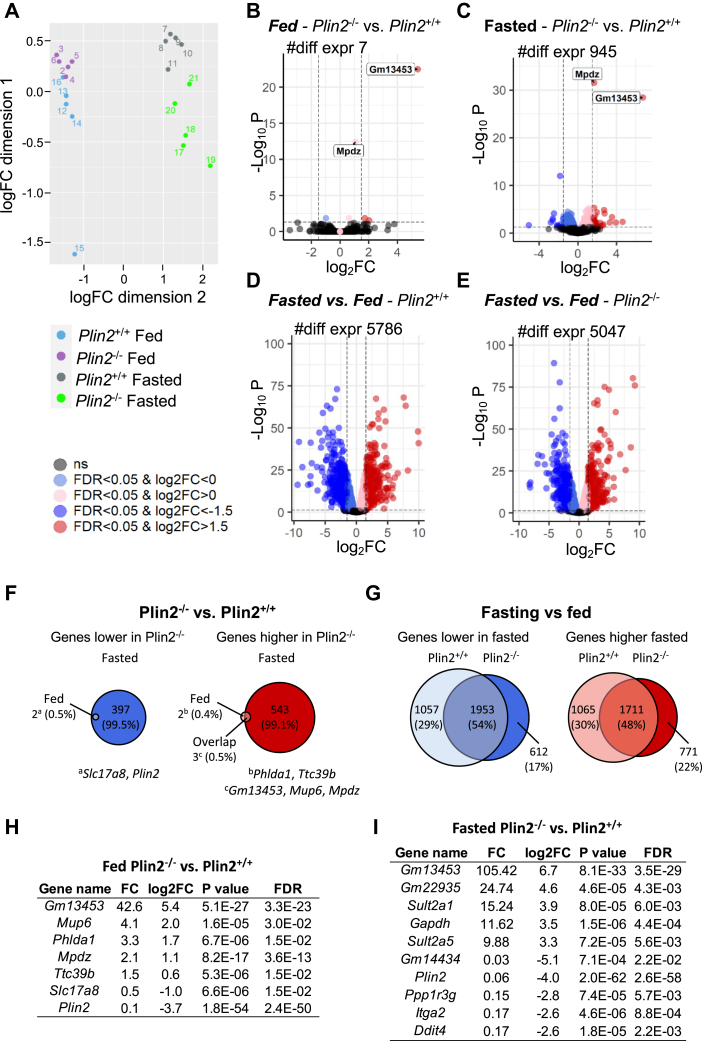
Global hepatic gene expression in fed and fasted *Plin2*^+/+^ and *Plin2*^−/−^ mice. Liver gene expression in female *Plin2*^+/+^ and *Plin2*^−/−^ mice (15 weeks) housed with ad libitum access to chow diet (fed) or fasted for 24 h (fasted). RNA-Seq was performed on five representative individuals per group (*n* = 5). A: Principal component analysis (PCA) plot of fed and fasted *Plin2*^+/+^ and *Plin2*^−/−^ mice. B: Volcano plot illustrating the number of individual transcripts with induced (red Log2FC >0.585 [50% increase] or light red Log2FC >0) or repressed (blue Log 2FC <−0.585 or light blue Log 2FC <0) expression levels in fed *Plin2*^−/−^ mice compared with fed *Plin2*^+/+^ mice. FDR (false discovery rate) <0.05. C: Transcripts with induced (red) or repressed (blue) expression levels in fasted *Plin2*^−/−^ mice compared with fasted *Plin2*^+/+^ mice. FDR <0.05. D: Transcripts with induced (red) or repressed (blue) expression levels in fasted compared with fed *Plin2*^+/+^ mice. FDR <0.05. E: Transcripts with induced (red) or repressed (blue) expression levels in fasted compared with fed *Plin2*^−/−^ mice. FDR <0.05. F: Diagram of genes that were lower (blue) or higher (red) expressed in *Plin2*^−/−^ mice compared with *Plin2*^+/+^ mice in the fed (light color) and fasted (dark color) states. G: Diagram of genes that were lower (blue) or higher (red) expressed in fasted versus fed *Plin2*^+/+^ mice (light color) and *Plin2*^−/−^ mice (dark color). H: List of transcripts that were differentially expressed in fed *Plin2*^−/−^ mice compared with fed *Plin2*^+/+^ mice. I: The top altered transcripts (five upregulated and five downregulated) that were differentially expressed in fasted *Plin2*^−/−^ and *Plin2*^+/+^ mice.

Next, we determined if lack of Plin2 expression influenced hepatic gene expression in the fed or fasted state. Livers from five representative individuals of fed or fasted *Plin2*^+/+^ and *Plin2*^−/−^ mice were subjected to RNA-Seq. Principal component analysis revealed substantial hepatic transcriptomic changes with fasting of *Plin2*^−/−^ and *Plin2*^+/+^ mice (Fig. 3A), with groups that clustered well together except for one distinctively different individual amongst fed *Plin2*^+/+^ mice. Differential gene expression analyses with this outlier included or excluded were similar, and the presented data include all individuals.

Detailed analyses of individual genes are illustrated in volcano plots, comparing *Plin2*^+/+^ and *Plin2*^−/−^ mice in fed and fasted states. Comparison of gene expression in fed *Plin2*^−/−^ versus *Plin2*^+/+^ mice (Fig. 3B, F and supplemental Table S2B) revealed only seven genes (Fig. 3H) that were differentially expressed (FDR <0.05). Two genes were downregulated (*Plin2* and *Slc17a8*), and five genes were upregulated (*Gm13453* [pseudogene], *Mpdz*, *Phlda1*, *Ttc39b*, and *Mup6*). As expected, fasting initiated a profound switch in hepatic gene expression. In total, 5,786 genes (3,010 down and 2,776 up) were differentially expressed (FDR <0.05) in fasted versus fed *Plin2*^+/+^ mice (Fig. 3D and supplemental Table S2D). A lower number of genes (5,047 genes, 2,565 down, and 2,482 up) were differentially expressed in fasted versus fed *Plin2*^−/−^ mice (Fig. 3E and supplemental Table S2E). Of note, of the total number of transcripts altered by fasting compared with feeding in *Plin2*^+/+^ and *Plin2*^−/−^ mice, around two-thirds were common, whereas around one-third were uniquely regulated for each genotype (Fig. 3G). These results suggest a moderately compromised fasting response in *Plin2*^−/−^ mice. Comparison of gene expression in fasted *Plin2*^−/−^ mice versus *Plin2*^+/+^ mice (Fig. 3C and supplemental Table S2C) revealed 945 differentially expressed genes (FDR <0.05; 399 genes were lower expressed and 546 genes higher expressed in *Plin2*^−/−^). The five genes that were strongest downregulated or upregulated in fasted *Plin2*^−/−^ mice versus *Plin2*^+/+^ mice are listed (Fig. 3I).

To identify pathways that were differently regulated upon fasting, we performed a KEGG pathway overrepresentation analysis with the 945 genes that were significantly increased or decreased in fasted *Plin2*^−/−^ mice compared with fasted *Plin2*^+/+^ mice. A majority of pathways that were increased in fasted *Plin2*^−/−^ mice were related to the immune system, in addition to cell adhesion and steroid biosynthesis (Table 2). Pathways identified as lower expressed in fasted *Plin2*^−/−^ mice were related to autophagy, insulin signaling, longevity, and apoptosis.

**Table 2:** KEGG pathway overrepresentation analysis on genes that were significantly increased or decreased in fasted *Plin2*^−/−^ mice compared with fasted *Plin2*^+/+^ mice (FDR < 0.05)

KEGG pathway	GeneRatio	Count	*P*	FDR (Adjusted *P*)
Genes that were higher in *Plin2*^−/−^ (top 15 pathways)				
Cell adhesion molecules	19/275	19	2.10E-06	2.97E-04
Viral myocarditis	13/275	13	1.82E-06	2.97E-04
Antigen processing and presentation	12/275	12	1.35E-05	1.27E-03
Intestinal immune network for IgA production	8/275	8	3.32E-05	2.35E-03
Asthma	6/275	6	7.34E-05	4.15E-03
Toxoplasmosis	12/275	12	1.02E-04	4.80E-03
Human T-cell leukemia virus 1 infection	19/275	19	1.70E-04	6.86E-03
Influenza A	15/275	15	2.01E-04	7.09E-03
Rheumatoid arthritis	10/275	10	2.50E-04	7.09E-03
Steroid biosynthesis	5/275	5	2.45E-04	7.09E-03
Th17 cell differentiation	11/275	11	2.83E-04	7.28E-03
Phagosome	15/275	15	3.49E-04	8.22E-03
Epstein-Barr virus infection	17/275	17	5.41E-04	9.00E-03
Hematopoietic cell lineage	10/275	10	4.71E-04	9.00E-03
Inflammatory bowel disease	8/275	8	4.74E-04	9.00E-03
Genes that were lower in *Plin2*^−/−^				
Autophagy—animal	13/132	13	1.07E-07	2.56E-05
Insulin signaling pathway	9/132	9	1.64E-04	1.97E-02
Longevity regulating pathway	7/132	7	2.93E-04	2.35E-02
Apoptosis	8/132	8	7.26E-04	4.36E-02

### Altered expression of immune-related genes in fasted *Plin2*^−/−^ mice

**Fig. 4:**
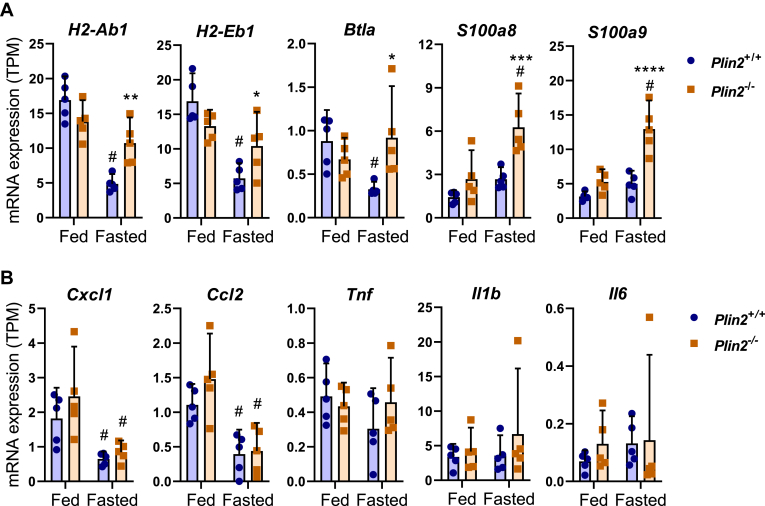
Hepatic expression of immune-related transcripts in fed and fasted *Plin2*^+/+^ and *Plin2*^−/−^ mice. Hepatic expression of selected mRNAs in female *Plin2*^+/+^ and *Plin2*^−/−^ mice (15 weeks) housed with ad libitum access to chow diet (fed) or fasted for 24 h (fasted). A: Expression of immune-related genes with altered expression in fasted *Plin2*^−/−^ mice (*H2-Ab1*, *H2-Eb1*, *Btla*, *S100a8*, and *S100a9*). B: Expression of typical hepatic inflammation marker genes (*Cxcl1*, *ccl2*, *Tnfa*, *Il1b*, and *Il6*). Statistical testing was done with two-way ANOVA and Tukey test for multiple comparisons. #*P* < 0.05 indicates statistical difference between fed and fasted conditions; ∗*P* < 0.05, ∗∗*P* < 0.01 and ∗∗∗*P* < 0.001 indicate differences between *Plin2*^+/+^ and *Plin2*^−/−^ mice with the same feeding condition.

Hepatic expression of the family member Plin5 has been shown to dampen inflammation during fasting (43). Since hepatic Plin5 expression is elevated (Fig. 1) and KEGG analysis revealed that immune-related pathways are differently regulated in fasted *Plin2*^−/−^ mice, we investigated the expression of immune-related genes in fed and fasted *Plin2*^+/+^ and *Plin2*^−/−^ mice. Several histocompatibility 2 genes (e.g., *H2-Ab1* and *H2-Eb1*) and the B and T lymphocyte-associated gene (*Btla*) were reduced by fasting in *Plin2*^+/+^ mice but to a lesser extent in *Plin2*^−/−^ mice (Fig. 4A). Conversely, the neutrophil-enriched calprotectin/S100 calcium-binding a8/a9 genes (*S100a1* and *S100a9*) were induced in fasted *Plin2*^−/−^ mice as compared with *Plin2*^+/+^ mice. Hepatic inflammatory markers such as *Cxcl1* and *Ccl2* were reduced by fasting but were not different between genotypes, whereas *Tnf*, *Il1b*, and *Il6* were not affected by fasting or removal of Plin2 (Fig. 4B). These expression patterns demonstrate that typical hepatic inflammation is unaffected in fed or fasted *Plin2*^−/−^ mice, which suggests that differences in the expression of immune-related genes are linked to alterations in hepatic immune cell populations.

### Unaltered autophagy but increased lipolysis in fasted *Plin2*^−/−^ mice

**Fig. 5:**
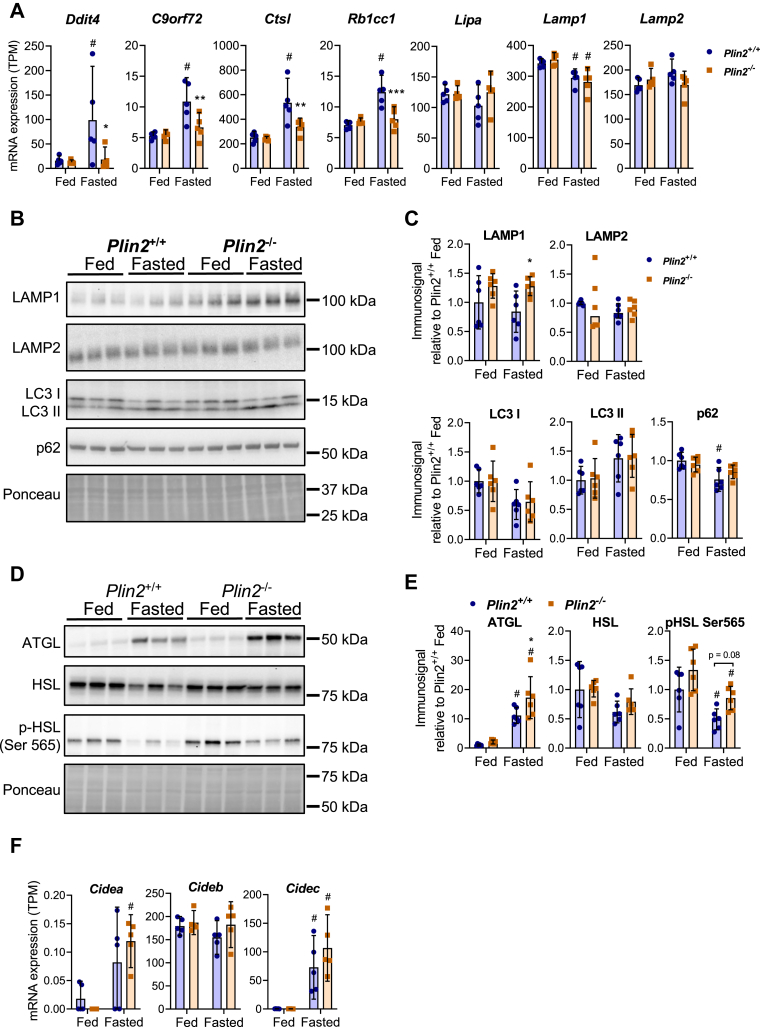
Hepatic expression of lipophagy markers and lipolytic enzymes in fed and fasted *Plin2*^+/+^ and *Plin2*^−/−^ mice. Expression of selected mRNAs and proteins in female *Plin2*^+/+^ and *Plin2*^−/−^ mice (15 weeks) housed with ad libitum access to chow diet (fed) or fasted for 24 h (fasted). A: Expression of autophagy-related genes with altered expression in fasted *Plin2*^−/−^ mice (*Ddit4*, *C9orf72*, *Ctsl*, *Rb1cc1*), lysosomal acid lipase (*Lipa*), and lysosomal markers *(Lamp1* and *Lamp2*). B: Expression of proteins involved in lipophagy and lysosomal function. Representative immunosignals of Lamp1 and Lamp2 (lysosomal), cytosolic (LC3-I) and lipidated (LC3-II) LC3 proteins, and the autophagy receptor (p62) in individual mice. Six representative animals were selected for each group (*n* = 6 per group). C: Relative quantification of hepatic Lamp1, Lamp2, LC3-I, LC3-II, and p62 protein immunosignals relative to expression in fed *Plin2*^+/+^ mice. D: Expression of proteins involved in lipolysis. Representative immunosignals of ATGL, HSL, and Ser 565 p-HSL (Ser 565) in individual mice. E: Relative quantification of hepatic ATGL, HSL, p-HSL (Ser 565) protein immunosignals relative to expression in fed *Plin2*^+/+^ mice. F: Expression of *Cidea. Cideb* and *Cidec* mRNAs in fed and fasted *Plin2*^+/+^ and *Plin2*^−/−^ mice. Statistical testing was done with two-way ANOVA and Tukey test for multiple comparisons. #*P* = 0.05 indicates the statistical difference between fed and fasted conditions. Data in graphs are shown as means ± 95% confidence interval.

The KEGG pathway analysis (Table 2) revealed that many of the genes that were lower expressed in fasted *Plin2*^−/−^ mice are involved in autophagy. Because altered lipophagy can potentially explain the depleted TAG stores and enlargement of LDs in fasted *Plin2*^−/−^ mice, we analyzed the hepatic expression of genes involved in lipophagy. Expression of several autophagy-related genes (*Ddit4*, *C9orf72*, *Ctsl*, and *Rb1cc1*) was induced by fasting in *Plin2*^+/+^ mice but not in fasted *Plin2*^−/−^ mice (Fig. 5A). Expression of lysosomal acid lipase (*Lipa*) and the lysosomal-associated membrane proteins 1 and 2 (*Lamp1* and *Lamp2*) were similarly expressed in fasted *Plin2*^+/+^ and *Plin2*^−/−^ mice. To evaluate lipophagy activity, we measured protein expression levels of Lamp1 and Lamp2, microtubule-associated protein 1A/1B- LC3, and the ubiquitin-binding protein p62. LAMP1 protein levels tended to be higher in fed *Plin2*^−/−^ mice and were higher in fasted *Plin2*^−/−^ mice compared with *Plin2*^+/+^ mice (Fig. 5B–C). Cytosolic LC3 I and membrane-bound and lipidated LC3 II were expressed at similar levels in fed and fasted *Plin2*^+/+^ and *Plin2*^−/−^ mice. Expression of the p62 protein remained unchanged, suggesting that hepatic lipophagy activity is unaffected by the removal of Plin2.

Plin2 is known to affect lipolysis and ATGL activity (51, 52). To determine if lipolysis is altered in *Plin2*^−/−^ mice, we analyzed the protein abundance of the lipases ATGL and HSL. ATGL protein levels increased with fasting, with higher expression levels in fasted *Plin2*^−/−^ mice compared with *Plin2*^+/+^ mice (Fig. 5D, E). Expression of HSL tended to be lower with fasting, whereas p-HSL (Ser 565) was reduced. There was an insignificant trend for higher levels of p-HSL (Ser 565) in fasted *Plin2*^−/−^ mice compared with fasted *Plin2*^+/+^ mice. Overall, increased levels of ATGL and a trend for higher levels of p-HSL (Ser 565) in *Plin2*^−/−^ mice compared with *Plin2*^+/+^ mice suggests that lipolytic activity is increased in fasted *Plin2*^−/−^ mice.

We then considered if differences in LD size morphology could be related to expression of cell death-inducing DNA fragmentation factor alpha-like effector (Cide) proteins, which are known regulators of LD fusion (53). Gene expression of *Cidea*, *Cideb*, and *Cidec* was unaltered between *Plin2*^+/+^ mice and *Plin2*^−/−^ mice, in the fed and fasted states (Fig. 5F). Expression levels were confirmed with RT-qPCR analysis (data not shown). Expression of *Cidea* was low, and while expression of *Cidea* and *Cideb* was unaffected by fasting, expression of *Cidec* mRNA was highly induced by fasting. These data do not suggest a simple mechanistic regulation of altered LD morphology in *Plin2*^−/−^ livers caused by differences in the expression of Cide proteins.

### Minor alterations in the expression of genes involved in FA oxidation and endoplasmic reticulum stress in fasted *Plin2*^−/−^ mice

**Fig. 6:**
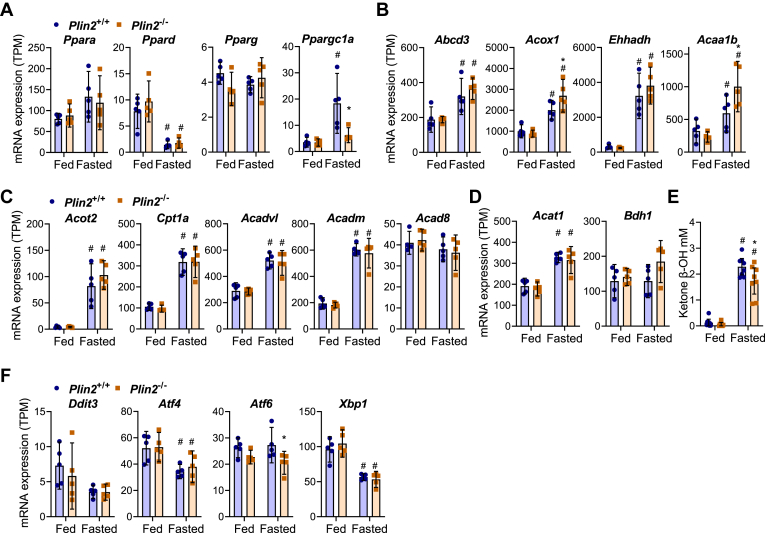
Hepatic expression of β-oxidation enzymes, ER stress markers, and inflammation in fed and fasted *Plin2*^+/+^ and *Plin2*^−/−^ mice. Hepatic gene expression and levels of plasma ketone bodies in female *Plin2*^+/+^ and *Plin2*^−/−^ mice (15 weeks) housed with ad libitum access to chow diet (fed) or fasted for 24 h (fasted). For all analyses (*n* = 8 per group). A: Expression of transcription factors activated by FAs (*Ppara*, *Ppard*, *Pparg*) and coactivator (*Ppargc1a*). B: Expression of peroxisomal β-oxidation genes (*Abcd3*, *Acox1*, *Ehhadh*, and *Acaa1b*). C: Expression of mitochondrial β-oxidation genes (*Acot2*, *Cpt1*, *Acadvl*, *Acadm*, and *Acad8*). D: Expression of ketogenic genes (*Acat1* and *Bdh1*). E: Plasma levels of the ketone body beta-hydroxybutyrate (BOH). F: Expression of the ER stress markers (*Ddit3*, *Atf4*, *Atf6*, and *Xbp1*). Statistical testing was done with two-way ANOVA and Tukey test for multiple comparisons. #*P* < 0.05 indicates statistical difference between fed and fasted conditions; ∗*P* < 0.05, ∗∗*P* < 0.01, and ∗∗∗*P* < 0.001 indicate difference between *Plin2*^+/+^ and *Plin2*^−/−^ mice with the same feeding condition.

We have shown previously that loss of Plin2 increases lipolysis and FA oxidation in myotubes (52). To determine if the same occurs in the liver of fasted *Plin2*^−/−^ mice, we analyzed expression of FA sensors, β-oxidation genes, and endoplasmic reticulum (ER) stress markers in our RNA-Seq dataset. As reported previously (54), fasting increased expression of peroxisome proliferator-activated receptor alpha (*Ppara*), an important hepatic FA sensor during fasting (Fig. 6A). Expression of *Ppara* and the family related *Ppard* and *Pparg* was similar in *Plin2*^+/+^ mice and *Plin2*^−/−^ mice, whereas expression of peroxisome proliferator-activated receptor gamma coactivator 1-alpha (*Ppargc1a*) was induced in fasted *Plin2*^+/+^ mice but not in fasted *Plin2*^−/−^ mice. Several mRNAs involved in peroxisomal β-oxidation (*Acox1* and *Acaa1b*) were expressed at somewhat higher levels in fasted *Plin2*^−/−^ mice compared with fasted *Plin2*^+/+^ mice (Fig. 6B). Expression of Pex14 protein, a peroxisomal marker (55), was unchanged in fasted *Plin2*^+/+^ compared with fasted *Plin2*^−/−^ mice (data not shown), suggesting a similar content of peroxisomes. Expression levels of mRNAs encoding enzymes involved in mitochondrial FA transport (*Acot2*, *Cpt1*) and mitochondrial β-oxidation (*Acadv1*, *Acadm*) increased at similar levels with fasting in *Plin2*^+/+^ and *Plin2*^−/−^ mice, whereas expression of *Acad8* mRNA, encoding an enzyme involved in the oxidation of both FAs and branch-chained amino acids, remained unaltered (Fig. 6C). Expression of *Acat1* and *Bdh1* mRNAs, which encode enzymes catalyzing the conversion of acetyl-CoA into acetoacetate and β-hydroxybutyrate, respectively, was similarly expressed in fasted *Plin2*^+/+^ and *Plin2*^−/−^ mice. Plasma levels of β-hydroxybutyrate were significantly increased with fasting but were less increased in fasted *Plin2*^−/−^ mice compared with *Plin2*^+/+^ mice (Fig. 6D).

Peroxisomal β-oxidation is known to generate free radicals, which potentially induce oxidative stress (55). Expression of mRNAs encoding proteins activated by ER stress (*Ddit3*, *Aft4*, *Atf6*, and *Xbp1*) was essentially similarly expressed in *Plin2*^+/+^ and *Plin2*^−/−^ mice, although some minor differences in expression levels were noted for some of these genes between fed and fasted mice (Fig. 6E). Based on these expression profiles, it seems unlikely that free radicals are induced sufficiently to cause ER stress in fasted *Plin2*^−/−^ mice.

### Comparable body weights, metabolic phenotypes, and glucose clearance in *Plin2*^+/+^ and *Plin2*^−/−^ mice

**Fig. 7:**
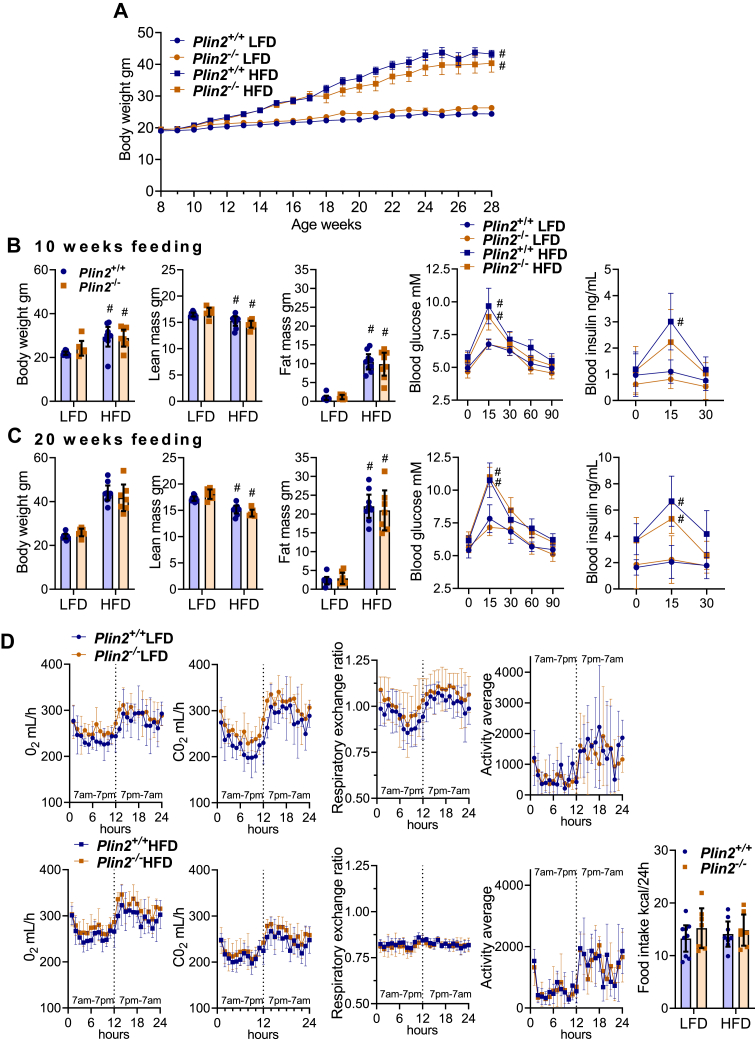
Body weight and composition, energy metabolism, and glucose clearance in *Plin2*^+/+^ and *Plin2*^−/−^ mice fed LFD or HFD. Female *Plin2*^+/+^ and *Plin2*^−/−^ mice were fed an LFD or an HFD from 8 weeks of age until 28 weeks of age. Mice were euthanized in the morning (8–10 AM) after 10 weeks of diet intervention (18 weeks of age) or 20 weeks of diet intervention (28 weeks of age). A: Body weights from the start of diet intervention (8 weeks of age) until the end of diet intervention (28 weeks of age). Number of individuals included in data points up to 10/20 weeks of diet intervention: *Plin2*^+/+^ LFD (*n* = 17/8), *Plin2*^+/+^ HFD (*n* = 19/9), *Plin2*^−/−^ LFD (*n* = 13/7), *Plin2*^−/−^ HFD (*n* = 17/7). B: Metabolic measurements after 10 weeks of diet intervention (18-week-old mice). Total body weights, lean mass, and fat mass at euthanasia. Whole blood glucose (0–120 min) and plasma insulin (0–30 min) levels after oral gavage of 1.5 g glucose/kg lean mass (OGTT) in 17-week-old mice. C: Metabolic measurements after 20 weeks of diet intervention (28-week-old mice). Total body weights, lean mass, and fat mass at euthanasia. Whole blood glucose (0–120 min) and plasma insulin (0–30 min) levels after oral gavage of 1.5 g glucose/kg lean mass (OGTT) in 27-week-old mice. D: Metabolic phenotyping of *Plin2*^+/+^ and *Plin2*^−/−^ mice fed LFD (upper raw) or HFD (lower raw) after 18 weeks of diet intervention. Oxygen consumption (O_2_, ml/h), carbon dioxide production (CO_2_, ml/h), respiratory exchange ratio (RER), activity levels (in the XY plane), mean activity levels in the light (7 AM–7 PM) and dark (7 PM–7 AM) phases, and daily calorie intake (kcal/24 h). Data in graphs are shown as means ± 95% confidence interval. Statistical testing was performed with two-way ANOVA and Tukey or Dunnett’s test for multiple comparisons. #*P* < 0.05 indicates the statistical difference between LFD and HFD for the same genotype.

The previous analyses demonstrate that hepatic Plin2 is required for normal lipid storage and lipid metabolism during fasting (metabolically; whole-body energy deficiency). Hepatic lipids are also increased with obesity (metabolically; whole body energy excess) (56). To investigate the role of Plin2 for hepatic LD formation and lipid metabolism in diet-induced obese mice, we exposed female *Plin2*^+/+^ and *Plin2*^−/−^ mice to LFD or HFD for up to 20 weeks. From around 7 weeks on the diet (15 weeks of age), the body weights of HFD-fed mice separated from the body weights of LFD-fed mice (Fig. 7A), but there were no differences in body weight curves between *Plin2*^+/+^ and *Plin2*^−/−^ mice on the same diet. After 10 weeks (Fig. 7B) and 20 weeks (Fig. 7C) of feeding, body composition measurements revealed that lean mass was slightly decreased with HFD compared with LFD, whereas fat mass was increased ∼10 g and ∼20 g with HFD, respectively. Hence, weight gain obtained with HFD was caused by fat accumulation. There were no differences in lean or fat mass between *Plin2*^+/+^ and *Plin2*^−/−^ mice (Fig. 7B–C). To evaluate glucose clearance, mice were exposed to an oral glucose bolus (1.5 g/kg lean mass). HFD-fed mice had increased blood glucose and insulin levels compared with LFD-fed mice, but there were no differences in glucose response or plasma insulin levels between *Plin2*^+/+^ and *Plin2*^−/−^ mice fed the diets for 10 weeks (Fig. 7B) or 20 weeks (Fig. 7C).

To investigate whole-body energy expenditure, *Plin2*^+/+^ and *Plin2*^−/−^ mice were monitored with indirect calorimetry after 9 and 18 weeks of diet intervention (data from 19 weeks of diet intervention are shown). O_2_ consumption was relatively similar in LFD- or HFD-fed mice. CO_2_ production and respiratory exchange ratio were different between LFD- and HFD-fed mice because of the consumption of primarily carbohydrates versus fat as the main energy substrates, respectively (Fig. 7D). There were no differences in O_2_ consumption, CO_2_ production, respiratory exchange ratio, activity levels, or food intake between *Plin2*^+/+^ and *Plin2*^−/−^ mice. We also measured the tissue weights of various organs in animals terminated after 10 and 20 weeks of diet intervention (18 and 28 weeks of age). Total body weights, as well as weights of epididymal and inguinal subcutaneous white adipose tissue depots, were elevated in HFD- compared with LFD-fed mice (Table 3). Liver, heart, and kidney weights were not affected by diets. There were no differences in organ weights between *Plin2*^+/+^ and *Plin2*^−/−^ mice (Table 3).

**Table 3:** Body weights and organ weights of *Plin2*^+/+^ and *Plin2*^−/−^ mice on LFD or HFD at 18 weeks (10 weeks on diet) and 28 weeks of age (20 weeks on diet)

Organs	LFD		HFD	
	*Plin2*^+/+^	*Plin2*^−/−^	*Plin2*^+/+^	*Plin2*^−/−^
18 weeks—females				
Body weight (g)	22.1 ± 1.0	23.8 ± 1.7	29.6 ± 4.5^a^	31.7 ± 6.0^a^
Liver (g)	0.99 ± 0.08	0.99 ± 0.08	1.12 ± 0.126	1.09 ± 0.15
Heart (g)	0.108 ± 0.024	0.119 ± 0.041	0.111 ± 0.033	0.118 ± 0.027
Kidney (g)	0.22 ± 0.02	0.24 ± 0.04	0.28 ± 0.06	0.28 ± 0.04
White adipose tissue gonadal (g)	0.35 ± 0.09	0.55 ± 0.27	1.38 ± 0.63^a^	1.7 ± 0.89^a^
White adipose tissue subcutaneous (g)	0.27 ± 0.09	0.36 ± 0.15	1.02 ± 0.49^a^	1.02 ± 0.50^a^
28 weeks—females				
Body weight (g)	24.4 ± 1.3	26.3 ± 2.1	43.3 ± 3.6^a^	40.4 ± 8.0^a^
Liver (g)	1.04 ± 0.08	1.11 ± 0.17	1.14 ± 0.41	1.13 ± 0.18
Heart (g)	0.100 ± 0.006	0.109 ± 0.008	0.118 ± 0.007	0.118 ± 0.022
Kidney (g)	0.23 ± 0.02	0.25 ± 0.02	0.28 ± 0.02	0.28 ± 0.03
White adipose tissue gonadal (g)	0.047 ± 0.11	0.73 ± 0.24	3.41 ± 0.39^a^	3.31 ± 1.02^a^
White adipose tissue subcutaneous (g)	0.36 ± 0.13	0.53 ± 0.20	2.30 ± 0.38^a^	2.10 ± 0.75^a^

Data are presented as means ± SD, *n* = 8–10.

a Indicates difference between LFD and HFD of the same genotype (*P* < 0.001).

### *Plin2*^−/−^ mice fed HFD were more resistant to hepatic TAG accumulation

**Fig. 8:**
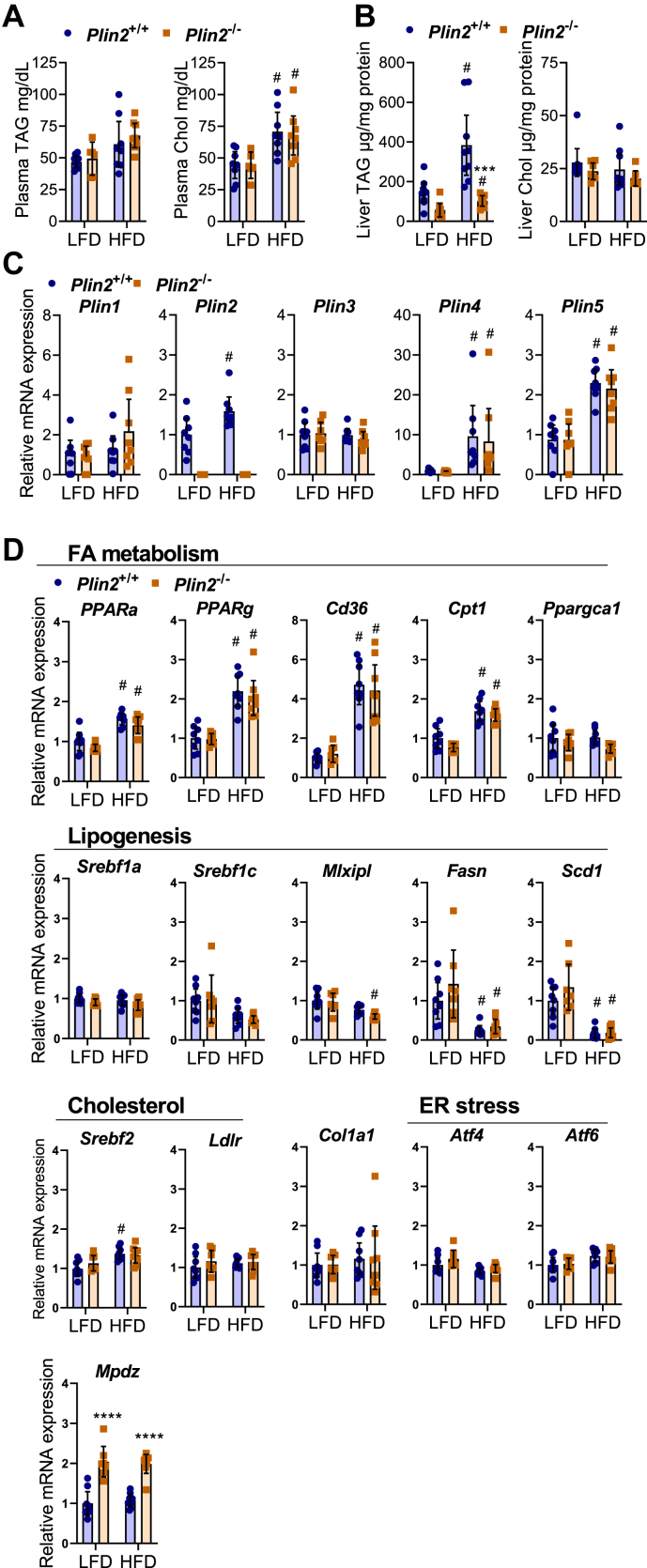
Lipid levels and hepatic mRNA expression in *Plin2*^+/+^ and *Plin2*^−/−^ mice fed LFD or HFD. Female *Plin2*^+/+^ and *Plin2*^−/−^ mice were fed an LFD or an HFD for 20 weeks (from 8 weeks of age until 28 weeks of age). Number of individuals: *Plin2*^+/+^ LFD (*n* = 8), *Plin2*^+/+^ HFD (*n* = 9), *Plin2*^−/−^ LFD (*n* = 7), and *Plin2*^−/−^ HFD (*n* = 7). A: Plasma TAGs and total Chol levels. B: Hepatic TAG and Chol levels. C: Hepatic expression of *Plin1*, *Plin2*, *Plin3*, *Plin4*, and *Plin5* mRNAs. D: Hepatic expression of mRNAs involved in FA metabolism (*Ppara*, *Pparg*, *Cd36*, *Cpt1*, *Ppargc1a*), lipogenesis (*Srebf1a*, *Srebf1c*, *Mlxipl*, *Fasn*, *Scd1*), Chol metabolism (*Srebf2*, *Ldlr*), fibrosis (*Col1a1*), ER stress (*Atf4* and *Atf6*), and expression of *Mpdz*. Statistical testing was done with two-way ANOVA and Tukey test for multiple comparisons. #*P* < 0.05 indicates the statistical difference between LFD and HFD for the same genotype. ∗∗∗*P* < 0.001 indicates difference between *Plin2*^+/+^ and *Plin2*^−/−^ mice receiving the same diet. Data in graphs are shown as means ± 95% confidence interval.

Although *Plin2*^−/−^ mice showed no distinguished phenotype with regard to adiposity, glucose clearance, or whole-body energy expenditure, we investigated if LFD- or HFD-fed *Plin2*^−/−^ mice had altered plasma or hepatic lipid content. Plasma TAG and total Chol levels were similar in *Plin2*^+/+^ and *Plin2*^−/−^ mice after 10 weeks (supplemental Fig. S3A) and 20 weeks of diet intervention (Fig. 8A), in line with no differences in these lipids between fed or fasted *Plin2*^+/+^ and *Plin2*^−/−^ mice. Hepatic TAG levels were similarly low with LFD feeding in *Plin2*^+/+^ and *Plin2*^−/−^ mice but were less induced in HFD-fed *Plin2*^−/−^ mice compared with *Plin2*^+/+^ mice after 10 weeks (supplemental Fig. S3) and 20 weeks of diet intervention (Fig. 8B). Hepatic total Chol levels were similar in LFD- or HFD-fed *Plin2*^+/+^ and *Plin2*^−/−^ mice. Next, we performed a detailed lipid analysis of liver tissue from five representative individuals per group of mice fed LFD and HFD for 10 weeks (18-week-old mice). The majority of TAG and DAG species measured were decreased in LFD- or HFD-fed *Plin2*^−/−^ mice compared with *Plin2*^+/+^ mice (supplemental Fig. S2). CE and other lipid species measured were essentially similar in *Plin2*^+/+^ and *Plin2*^−/−^ mice fed the same diet (supplemental Fig. S2).

### Hepatic expression of metabolic genes is unaltered in HFD-fed *Plin2*^−/−^ mice

Others have reported differences in weight gain and altered hepatic expression of metabolic genes in *Plin2*^−/−^ mice fed obesogenic diets (38, 39, 57). We therefore measured the hepatic expression of Plins and metabolic genes in LFD- and HFD-fed *Plin2*^+/+^ and *Plin2*^−/−^ mice. Expression of *Plin2*, *Plin4*, and *Plin5* mRNAs increased with HFD feeding compared with LFD after 20 weeks of diet intervention (Fig. 8C). This dietary effect was less pronounced after 10 weeks of feeding (supplemental Fig. S3B). Except for loss of *Plin2* expression in *Plin2*^−/−^ mice, expression levels of other Plins were similar in *Plin2*^+/+^ and *Plin2*^−/−^ mice with both diets, suggesting there were no transcriptional compensations in Plin expression upon loss of Plin2. Furthermore, we observed no differences in expression levels between *Plin2*^+/+^ and *Plin2*^−/−^ mice for genes involved in FA metabolism (*Ppara*, *Pparg*, *Cd36*, *Cpt1*, *Ppargc1a*), lipogenesis (*Srebf1*, *Mlxipl*, *Fasn*, *Scd1*), Chol metabolism and uptake (*Srebf2* and *Ldlr*), fibrosis (*Col1a1*), or ER stress (*Atf4* and *Atf6*) (Fig. 8D). *Mpdz* mRNA was upregulated ∼2-fold in *Plin2*^−/−^ mice compared with *Plin2*^+/+^ mice but was unaffected by diet (Fig. 8D).

## Discussion

Studies to characterize the role of hepatic Plin2 have primarily focused on its role in obesity (38, 39) and the development of nonalcoholic fatty liver disease caused by surplus energy intake (10, 39, 58, 59). In the current study, we compared the significance of Plin2 expression for hepatic lipid storage in mice subjected to energy deficiency (fasting) or energy surplus (HFD feeding). Fasting for 24 h resulted in a >10-fold increase in hepatic TAG levels in contrast to a ∼3-fold increase in hepatic TAG levels with 20 weeks of HFD feeding. Hepatic TAG levels in *Plin2*^−/−^ mice were reduced by both fasting and HFD feeding, yet with different consequences upon loss of Plin2. Hepatic LD morphology (result not shown) and expression of glucose and FA-oxidizing genes were unaltered in *Plin2*^−/−^ mice given an HFD. In contrast, LD morphology was drastically altered upon fasting, accompanied by somewhat elevated expression of immune-related genes and genes involved in autophagy and peroxisomal FA oxidative pathways in *Plin2*^−/−^ mice compared with *Plin2*^+/+^ mice. Taken together, our results show that hepatic expression of Plin2 is more important for coating of hepatic LDs formed upon fasting compared with LDs accumulated as a consequence of diet-induced hepatic steatosis.

A prominent phenotype in our studies was reduced hepatic steatosis in both fasted or HFD-fed *Plin2*^−/−^ mice. The first generated *Plin2*-null model (with disruption of *Plin2* exons 2–3) also showed reduced hepatic TAG levels (10), but this model expresses a shortened Plin2 isoform that might confound this observation (60). The same hepatic phenotype has later been reported in a different *Plin2*-null model (disrupting *Plin2* exon 5) (39), with siRNA silencing of *Plin2* in diet-induced obese mice (41) or genetically obese leptin-deficient (ob/ob) mice (61), or in mice with liver-specific loss of *Plin2* fed a methionine-choline deficient diet (40). Similarly, we also observed reduced hepatic TAG in our *Plin2* model (disruption of exons 4–6, (44)), albeit not in mice fed diets with low-fat content, which had low hepatic TAG levels. A less pronounced hepatic phenotype in nonsteatotic livers may be explained by the cellular location of Plin2 and distinct TAG reservoirs in hepatocytes. Hepatocytes can incorporate TAG in cytoplasmic LDs and membrane-enclosed VLDL particles. Plin2 binds to cytoplasmic LDs (11) and is actively degraded by proteasomes when not bound to LDs (12, 13). Although the presence of Plin2 may balance transfer of TAG toward LD versus VLDL pools (37, 59), VLDL is synthesized in the ER lumen and the Golgi apparatus and has no direct contact with Plin2 or LDs. Hepatocytes with low TAG levels are therefore expected to direct most TAG toward VLDL, with low dependence on Plin2 expression. In contrast, HFD or fasting elevate TAG incorporation into cytosolic LD in hepatocytes, opening for more manifested effects upon Plin2 removal. Our findings that lack of Plin2 has a limited effect on hepatic gene transcription in fed hepatocytes (low TAG) versus considerable effect in fasted hepatocytes (elevated TAG) imply that phenotypical alterations of Plin2 removal are primarily linked to the cellular need for storage of TAG.

It is well established that Plin2 overexpression increases LD accumulation (37, 42) and that loss of Plin2 reduces LD accumulation in various cells (10, 40, 41, 59, 61). It is, however, unclear if loss of Plin2 primarily affects LD size or LD numbers, and if loss of Plin2 enhances LD degradation primarily by altering rates of lipolysis or lipophagy. The first study demonstrating that removal of nonadipose Plin proteins could affect LD morphology was performed in hepatocyte-like cells (AML12), where siRNA silencing of Plin3 reduced LD size (Plin2 overexpression), silencing of *Plin2* had no effect, whereas *Plin2* and *Plin3* silencing resulted in few but enlarged LDs (62). Studies in animals have reported enlarged hepatic LDs in *Plin2*^−/−^ mice crossed into obese *Lep*^−/−^ deficient mice (59), and in adrenals of *Plin2*^−/−^ mice (44), yet with diverse effects on LD size and total LD mass. Removal of Plin2 results in increased adrenal CE levels and LD size (44), whereas hepatic TAG levels and total LD content are reduced despite minor elevations in hepatic CE levels in fasted or HFD-fed *Plin2*^−/−^ mice. Reduced LD content has also been reported in foam cells of *Plin2*^−/−^ mice in an atherogenic *ApoE*^−/−^ background (63), but lipid species and LD sizes were not evaluated. We now demonstrate that perhaps the most radical increase in hepatic LD size occurs in fasted *Plin2*^−/−^ mice, although the mechanisms involved in this phenotype are unclear and may be beyond the role of Plin2 as a protector against lipases. We observed increased levels of Plin3 and Plin5 proteins in fasted *Plin2*^−/−^ mice, implying that these Plin proteins compensate at the LD surface in the lack of Plin2. Such a shift in LD protein coating is likely to alter the recruitment of lipases and other proteins. Attempts to isolate LDs and reproducibly compare the hepatic LD proteome and lipidome in fasted *Plin2*^+/+^ and *Plin2*^−/−^ mice have proven difficult because of the extreme differences in size and buoyancy for those LD populations. However, whole hepatic cell lysates of fed *Plin2*^−/−^ mice had increased levels of the most abundant PC and PCO lipid species as well as increased levels of some of the PE and PS lipids upon fasting. It has been suggested that the cylindrical shape of PC facilitates good coverage of the LD surface and promotes LD stability (64, 65), whereas PE forms cone-shaped structures resulting in LD packing defects when present in excess (64). It is possible that increased hepatic levels of PC and PCO species in fed *Plin2*^−/−^ mice accumulate on LDs and help to stabilize the LD surface, whereas the increased levels of PE species in fasted *Plin2*^−/−^ mice cause LD packaging defects. Detailed experiments on isolated LDs will be needed to determine if the lack of Plin2 affects the LD phospholipid layer and contributes to the abnormal LD morphology in fasted *Plin2*^−/−^ mice.

The ability of various Plins to protect LDs seems influenced by the type of neutral lipids stored in the core (66). Emerging data suggest that TAG reservoirs are more strongly affected in *Plin2*^−/−^ mice compared with CE reservoirs that tend to be maintained or may even be enhanced (44). ATGL is the key regulator of lipolytic TAG degradation in many metabolic tissues, with activity levels influenced by expressed Plins (67). Overexpression of Plin2 in fibroblasts reduces ATGL activity and preserves TAG stores (52), whereas ATGL activity is enhanced and TAG stores depleted in *Plin2*^−/−^ myotubes (53). A limitation with these in vitro cell culture studies is the insignificant Plin5 expression (53, 68) which does not fully recapitulate the LD proteomes of the same cell types in vivo. Such limitations are especially relevant when analyzing Plin2 function in the liver, given that Plin5 binds and regulates the activity of ATGL (69), steatosis is exacerbated in *Pnpla2* (ATGL)^−/−^ mice (70), and as shown in this study, Plin5 is overexpressed in *Plin2*^−/−^ livers. The current literature supports the notion that hepatic Plin2 protects LDs against ATGL activity. Of note, we observed increased hepatic expression of Plin5 and ATGL in fasted *Plin2*^−/−^ mice, which suggests that elevated lipolysis is involved in the depletion of hepatic TAG in *Plin2*^−/−^ mice. Another possible mechanism involved is lipophagy, a form of autophagy where whole or partial LDs are engulfed by phagosomes and neutral lipids are degraded by lysosomal lipases and other enzymes (71). We observed slightly increased hepatic LAMP1 expression in fasted *Plin2*^−/−^ mice compared with the *Plin2*^+/+^ mice but similar levels of LC isoforms and p62, suggesting that altered lipophagy activity likely not contribute to reduced hepatic TAG levels in female *Plin2*^−/−^ mice. These results differ from a previous study reporting increased hepatic autophagy in fed male *Plin2*^−/−^ mice (51). It is unclear if these discrepancies are caused by different experimental conditions or gender-specific consequences of Plin2 loss. Of note, we previously observed increased content of the lipidated LC3 II and LAMP1/2 proteins in the adrenals of fed female *Plin2*^−/−^ mice (44), but these alterations may occur because of the accumulation of ceroid-like structures in some apoptotic/dead cortical cells and may not necessarily be a sign of altered autophagy in functional cortical cells. In summary, although ATGL seems involved, additional studies will be needed to fully define mechanisms involved in the depletion of hepatic TAG reservoirs in *Plin2*^−/−^ mice upon fasting.

Secondary systemic consequences of reduced hepatic LD accumulation in *Plin2*^−/−^ mice have been reported by independent laboratories. Improved insulin sensitivity has been observed with hepatic siRNA silencing of *Plin2* in HFD-fed (61) and alcohol-fed mice (72), in *Plin2*^−/−^ mice crossed into obese *Lep*^−/−^ deficient mice (59), and with HFD or Western diet feeding of mice with global or hepatic-specific *Plin2* deletion (38, 39, 58). In contrast to these reports, the initial characterization of *Plin2*^−/−^ mice did not report effects on insulin sensitivity (10). In addition, overexpression of *Plin2* in muscle seems to increase insulin sensitivity in parallel with enhanced LD accumulation (42), which opens for tissue-specific effects upon Plin2 deletion. Contradictory to most studies mentioned previously performed in males, we observed similar body weights, organ weights, body composition, glucose tolerance, energy expenditure, and hepatic expression of metabolic genes between female *Plin2*^+/+^ mice and *Plin2*^−/−^ mice fed an HFD for up to 20 weeks. Although gender differences could potentially explain divergent effects on insulin sensitivity in this case, we have not been able to replicate gross differences in weight gain between male *Plin2*^+/+^ and *Plin2*^−/−^ mice given an obesogenic Western-like diet (result not shown). Whole-body insulin sensitivity is determined by several organs, and inconsistent effects on glucose clearance between studies can potentially be caused by the usage of models with global or hepatocyte-specific deletion of Plin2. However, effects on body weight gain are inconsistently reported in many of the dietary studies performed in Plin2 models (38, 39, 58), suggesting reproducibility issues. Importantly, parameters reported to differ between *Plin2*^+/+^ and *Plin2*^−/−^ mice with obesogenic diet feeding in previous studies, inducing improved insulin sensitivity, are paralleled by a lack of weight gain in *Plin2*^−/−^ mice (38, 39, 58). The same metabolic differences will in large be observed between lean versus obese mice independent of Plin2 removal (72). Hence, it will be necessary to conduct new diet interventions to elucidate why effects on weight gain differ between laboratories and Plin2-null models used, and whether phenotypic differences observed in diet interventions are caused by Plin2 deletion in the liver or systemic or unknown confounding factors affecting body weight gain under certain conditions. Such confounding factors could perhaps be related to microflora or the immune system. Our diet intervention was performed in healthy mice absent from all pathogens according to FELASA guidelines. Details on animal health were not described in the other studies. Another possibility is differences in back-crossing between the models examined. We observed ∼2-fold elevated expression of multiple PDZ domain crumbs cell polarity complex component (*Mpdz*) mRNA in *Plin2*^−/−^ mice independent of dietary conditions. Our Plin2 model was generated in ES cells of a 129 background and has been backcrossed into C57BL/6N for at least 10 generations. The *Mpdz* loci is located ∼5 Mbp downstream of *Plin2* on mouse chromosome 4 (National Center for Biotechnology Information, assembly #NC_000070.7), and this whole chromosomal region is therefore unlikely to be backcrossed but remain as a 129 genome. Hence, it cannot be determined if the altered expression of the *Mpdz* gene in our model is caused by Plin2 loss or SNP variability in the *Mpdz* gene region between the 129 genome and C57BL/6 strains that affects expression levels of the *Mpdz* gene.

To conclude, our study examined hepatic effects upon deletion of *Plin2* in female mice. Fasted female *Plin2*^−/−^ mice had altered hepatic TAG and CE levels, abnormal LD morphology, increased protein levels of Plin3, Plin5, and ATGL, and grossly altered hepatic gene expression compared with fasted *Plin2*^+/+^ mice. In contrast, female *Plin2*^−/−^ mice fed an HFD for up to 20 weeks had reduced hepatic TAG, but otherwise, no differences in body weight gain, tissue weights, body composition, glucose tolerance, energy expenditure, or hepatic expression of metabolic genes compared with *Plin2*^+/+^ mice were observed. Our analysis suggests that Plin2 is more important for coating of hepatic LDs during fasting as compared with obesity-induced hepatic steatosis.

## Data availability

Data that support the findings of this study are available from the corresponding author upon reasonable request.

## Supplemental data

This article contains supplemental data.

## Conflict of interest

The authors declare that they have no conflicts of interest with the contents of this article.

## Supplemental Data

Supplemetary material
