# Supplementary material for: Altered hepatic lipid droplet morphology and lipid metabolism in fasted Plin2-null mice
Source: J Lipid Res. 2023 Oct 14;64(12):100461. doi: 10.1016/j.jlr.2023.100461 (PMC10716011; doi:10.1016/j.jlr.2023.100461)
Supplement: Supplemetary material [file mmc1.pdf]

Supplemental Fig. S1

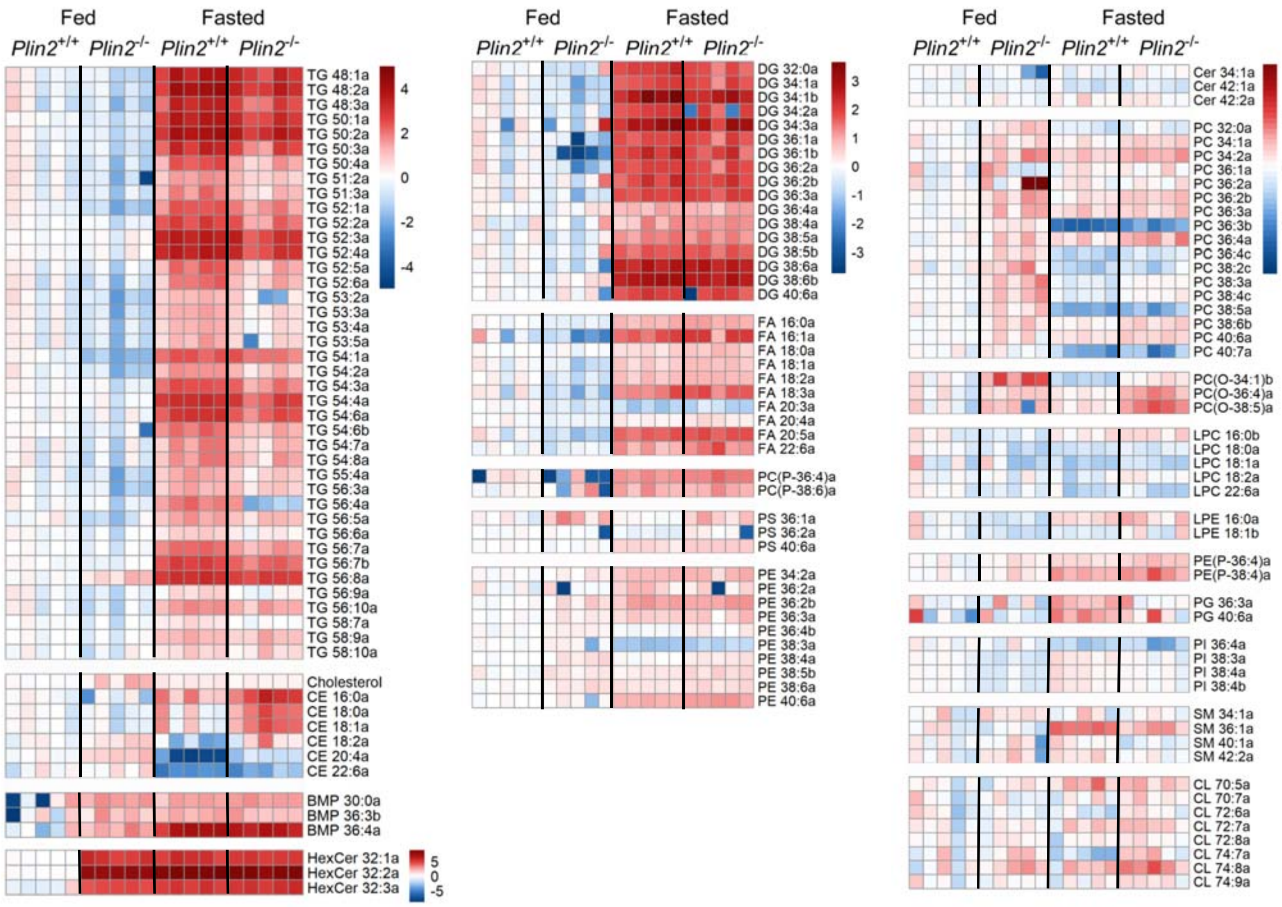

Supplemental Fig. S1.
Detailed lipid analysis of hepatic lipid species in *Plin2*$^{+/+}$ and *Plin2*$^{-/-}$ mice fed or fasted for 24 h (n=5 pr group). Data is presented as log fold-change (log2FC) relative to the mean for fed *Plin2*$^{+/+}$ mice. Measured lipid classes:
cardiolipin (CL), ceramide (Cer), cholesterol, cholesteryl ester (CE), diacylglycerol (DG), ether-linked phosphatidylcholine (PCO), free fatty acid (FA), hexosylceramide (HexCer), monoacylglycero-phosphate (BMP), lysophosphatidylcholine (LPC), lysophosphatidyl-ethanolamine (LPE), phosphatidylcholine (PC), phosphatidylcholine plasmalogen PCP, phosphatidylglycerol (PG), sphingomyelin (SM), phosphatidylethanolamine (PE), phosphatidylethanolamine plasmalogen (PEP), phosphatidylglycerol (PG), phosphatidylinositol (PI), phosphatidylserine (PS), and triglyceride (TG).

Supplemental Fig. S2

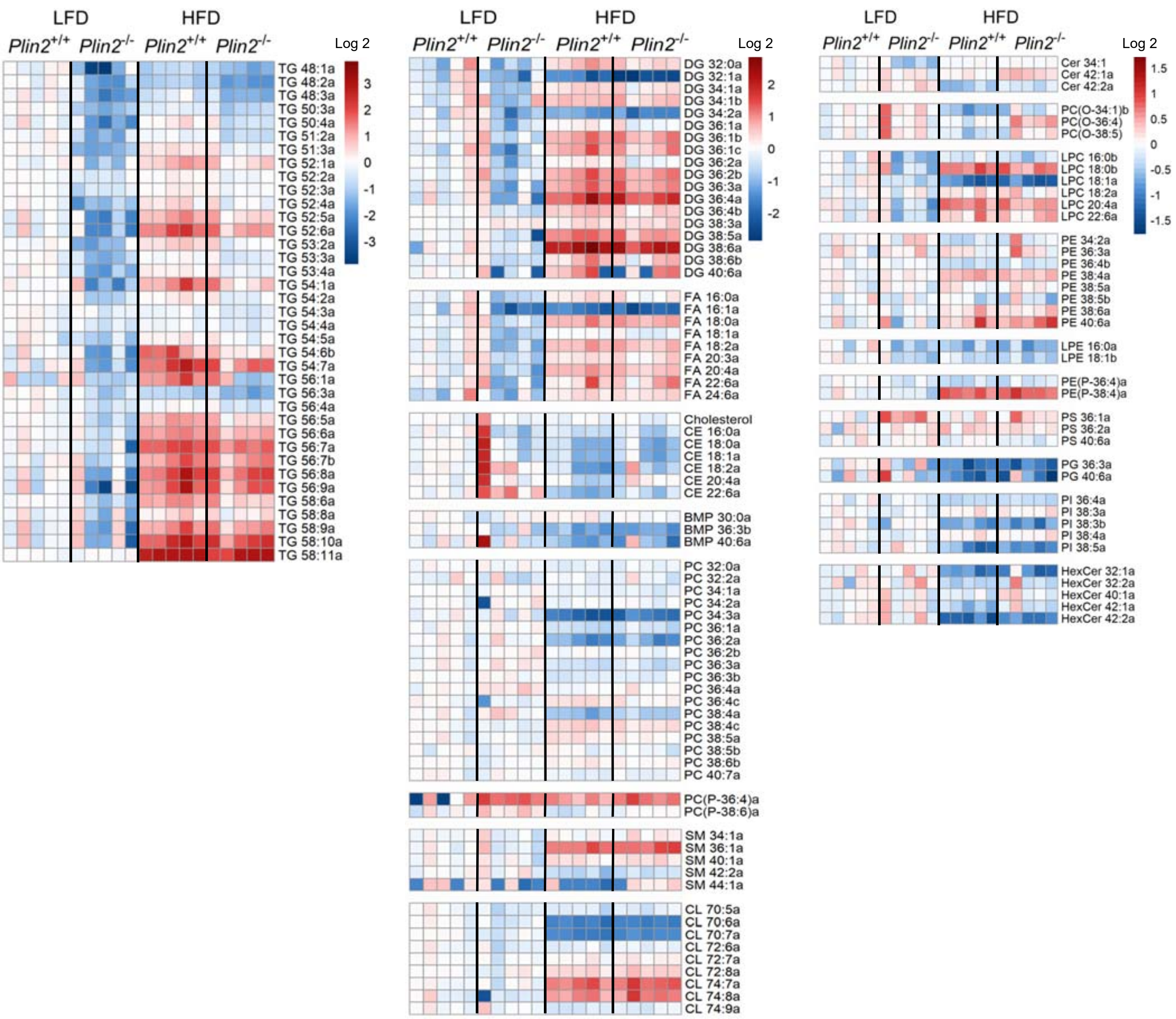

Supplemental Fig. S2.
Detailed lipid analysis of hepatic lipid species in *Plin2*$^{+/+}$ and *Plin2*$^{-/-}$ mice fed a low-fat diet (LFD or a high fat diet (HFD) for 10 weeks until 18 weeks of age (n=5 pr group). Data is presented as log fold-change (log2FC) relative to the mean for LFD fed *Plin2*$^{+/+}$ mice.
Measured lipid classes:
cardiolipin (CL), ceramide (Cer), cholesterol, cholesteryl ester (CE), diacylglycerol (DG), ether-linked phosphatidylcholine (PCO), free fatty acid (FA), hexosylceramide (HexCer), lysophosphatidylcholine (LPC), lysophosphatidylethanolamine (LPE), monoacylglycero-phosphate (BMP), phosphatidylcholine (PC), phosphatidylcholine plasmalogen PCP, phosphatidylethanolamine (PE), phosphatidylethanolamine plasmalogen (PEP), phosphatidylglycerol (PG), phosphatidylinositol (PI), phosphatidylserine (PS), sphingomyelin (SM), and triglycerides (TG).

Supplemental Fig. S3

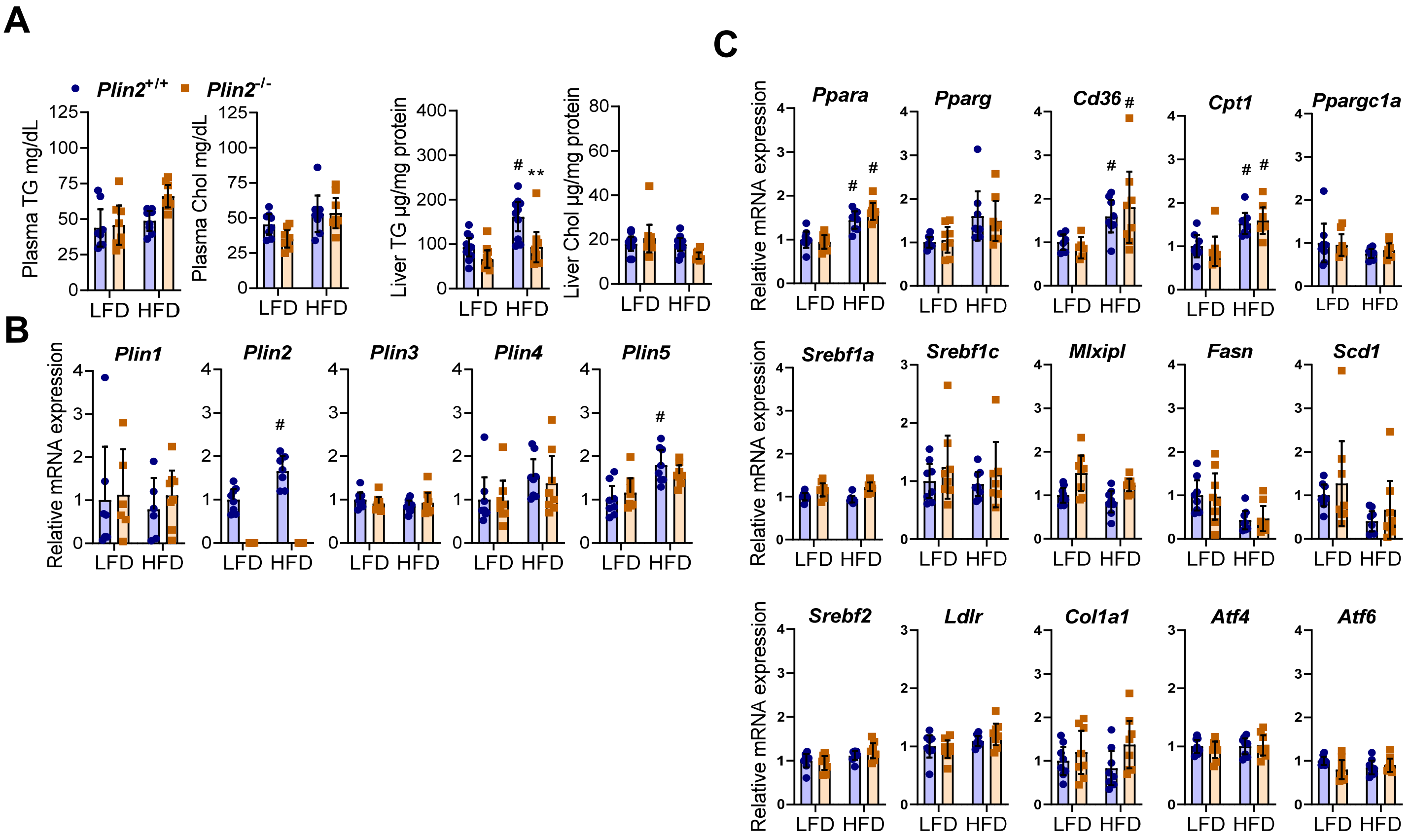

Supplemental Fig. S3. Lipid levels and hepatic mRNA expression in mice fed LFD or HFD for 10 weeks

Female *Plin2*$^{+/+}$ and *Plin2*$^{-/-}$ mice were fed low-fat diet (LFD) or high-fat diet (HFD) for 10 weeks from 8 weeks of age until 18 weeks of age. Number of individuals: *Plin2*$^{+/+}$ LFD (n=8), *Plin2*$^{+/+}$ HFD (n=10), *Plin2*$^{-/-}$ LFD (n=6), *Plin2*$^{-/-}$ HFD (n=10).
A) Plasma and hepatic triacylglycerides (TAG) and total cholesterol (Chol) levels (n=8).
B) Hepatic expression of *Plin1*, *Plin2*, *Plin3*, *Plin4* and *Plin5* mRNAs (n=8 pr group).
C) Hepatic expression of mRNAs involved in FA metabolism (*Ppara*, *Pparg*, *Cd36*, *Cpt1*, *Ppargc1a*), lipogenesis (*Srebf1a*, *Srebf1c*, *ChREBP*, *Fasn*, *Scd1*), cholesterol metabolism (*Srebf2*, *Ldlr*), fibrosis (*Col1a1*), and ER stress (*Atf4* and *Atf6*) (n=8 pr group).
Statistical testing was done with two-way ANOVA and Tuckey test for multiple comparisons. # indicate statistical difference between fed and fasted mice of the same genotype. ***p < 0.001 indicate difference between *Plin2*$^{+/+}$ and *Plin2*$^{-/-}$ mice under the same feeding condition. Data in graphs are shown as means ± 95% confidence interval.

Supplemental Tables

Supplemental Table 1. Primers used for RT-qPCR

Gene name	Accession	Forward primer	Reverse primer	Product size	Intron length
Atf4	NM_009716.3	TGGATGATGGCTTGGCCAGT	TTCTCCAACATCCAATCTGTCCC	112	2
Atf6	NM_001081304.1	TGCCACCAGAAGTATGGGTTC	ACTGACAAGCAGACTCTCGG	83	15287
Cd36	NM_001159557.1	AGGCATTCTCATGCCAGTCG	TGTACACAGTGGTGCCTGTT	119	8174
Col1a1	NM_007742.4	CTGACGCATGGCCAAGAAGAC	CCTCGGGTTTCCACGTCTCA	88	1465
Cpt1a	NM_013495.2	CCCAGCTGTCAAAGATACCGT	GCTGTCATGCGTTGGAAGTC	84	3807
Fasn	NM_007988.3	CTTCGGCTGCTGTTGGAAGTC	GTGTTCGTTCCTCGGAGTGAG	80	1005
Ldlr	NM_010700.3	GACTGCAAGGACATGAGCGA	TGTCCAAGCTGATGCACTCC	103	1860
Mlxipl	NM_021455.4	TGCAGCCCAGCCTAGATGAC	AGCTGGGGGACTCTATGTAGTT	102	4495
Mpdz	NM_001305286.1	GACTCACACACAGGCGGTTA	TCTTGCTGATGACCCGTGAC	105	563
Plin1	NM_001113471.1	ACCTGGAGGAAAAGATCCCG	TTCGAAGGCGGGTAGAGATG	87	1316
Plin2	NM_007408.3	GGGCTAGACAGGATGGAGGA	CACATCCTTCGCCCCAGTTA	99	2215
Plin3	NM_025836.3	CGAAGCTCAAGCTGCTATGG	TCACCATCCCATACGTGGAAC	98	1147
Plin4	NM_020568.3	ACCAACTCACAGATGGCAGG	AGGCATCTTCACTGCTGGTC	109	1213
Plin5	NM_001077348.1	GGTGAAGACACCACCCTAGC	CCACCACTCGATTCACCACA	115	568
Ppara	NM_001113418.1	ACTACGGAGTTCACGCATGT	GTCGTACACCAGCTTCAGCC	74	1710
Pparg	NM_001127330.1	TTGCTGTGGGGATGTCTCAC	AACAGCTTCTCCTTCTCGGC	70	12003
Ppard	NM_011145.3	ACATGGAATGTCGGGTGTGC	CGAGCTTCATGCGGATTGTC	108	1590
Ppargc1a	NM_008904.2	AGTCCCATACACAACCGCAG	CCCTTGGGGTCATTTGGTGA	94	4355
Scd1	NM_009127.4	GAGGCGAGCAACTGACTATC	GGTGGTCGTGTAAGAACTGG	71	625
Srebf1a	NM_001313979.1	GGCCGAGATGTGCGAACTG	GTTGTTGATGAGCTGGAGCATGT	70	13195
Srebf1c	XM_006532716.2	GGAGCCATGGATTGCACATTT	CAGCATAGGGGGCGTCAAA	91	3078
Srebf2	NM_033218.1	TGACTCTCGGGGACATCGAC	CACCTCCAGGGAAGGAGCTA	105	22247
